# The phytoplasma effector, phyllogen, structurally mimics host plant MADS transcription factors

**DOI:** 10.1016/j.jbc.2026.113449

**Published:** 2026-08-13

**Authors:** Antonin Galien, Stephanie Hutin, Sarah Le Hir, Michel Paul, Pascal Albanese, Sylvie Kieffer-Jaquinod, Audrey Guillotin, Nozomu Iwabuchi, Kensaku Maejima, Max H. Nanao, Mark D. Tully, Veronique Hugouvieux, Chloe Zubieta

**Affiliations:** 1 Laboratoire de Physiologie Cellulaire et Végétale, Université Grenoble-Alpes, CNRS, CEA, INRAE, IRIG-DBSCI, Grenoble, France; 2 Laboratoire Biosciences et Bioingénierie pour la Santé, Université Grenoble-Alpes, CEA, INSERM, UA13 BGE, CNRS, Grenoble, France; 3 Department of Agricultural and Environmental Biology, Graduate School of Agricultural and Life Sciences, The University of Tokyo, Tokyo, Japan; 4 MXIS Group, European Synchrotron Radiation Facility, Grenoble, France

**Keywords:** MADS transcription factors, flower development, host–pathogen interactions, phyllogen, phytoplasmas, floral organ identity

## Abstract

Phytoplasmas are obligate plant pathogens that include species causing severe developmental abnormalities in diverse plant families. Phytoplasma infections may result in dwarfism, witches’ broom, virescence, and phyllody, the conversion of reproductive organs into leaf-like structures. Phyllody is due to the activity of phytoplasma effector proteins, called phyllogens, that interact with the host MADS transcription factor (MTF) network. This interaction was believed to be specific for members of the AP1/SEP/AGL6 superclade of tetrameric MTFs, key regulators of floral meristem identity and organogenesis. Here, we determined the molecular and atomic basis for the interaction between the phyllogen from onions yellow phytoplasma (PHYL_OY_) and the floral MTFs, APETALA1 (AP1), SEPALLATA3 (SEP3), and AGAMOUS (AG) using biochemical, structural, and genetic experiments. The DNA-binding patterns of homo and heteromeric MTFs with PHYL_OY_ are shown using electrophoretic mobility shift assays, revealing broader interaction patterns between PHYL_OY_ and MTFs that depend on highly conserved hydrophobic residues. Point mutations of these residues allowed gain or loss of interaction between MTFs and PHYL_OY._ Expressing PHYL_OY_ under the control of the *SEP3* and *AP1* promoter in Arabidopsis further demonstrated whorl-specific phenotypes, recapitulating the *in vitro* interaction and DNA-binding patterns. These findings reveal how PHYL_OY_ targets specific MTFs through structural mimicry, resulting in impaired MTF tetramer formation and homeotic conversion of floral organs due to loss of active MTF tetramers.

Phytoplasmas (class *Mollicutes*, genus *Candidatus* Phytoplasma) are obligate plant pathogenic bacteria lacking a cell wall that are transmitted by phloem-feeding insects (1, 2). Symptoms of phytoplasma infection in plants include dwarfism, witches’ broom (abnormal shoot clusters and stem branching), virescence (greening of floral organs), and phyllody (conversion of floral organs into leaf-like structures) (2, 3, 4, 5). Phytoplasma infections have been identified in members of the Apiaceae, Asteraceae, Cucurbitaceae, Fabaceae, Malvaceae, Poaceae, Vitaceae, and Solanaceae families, causing significant damage to these plant populations (6, 7). Due to their ability to infect a wide variety of wild and domesticated plant species coupled with the resulting economic and ecological loss, understanding phytoplasma–host interactions is under active investigation. These studies are particularly topical, as climate change has altered and expanded insect vector habitats, resulting in phytoplasma infections at more northern latitudes (1, 6, 8, 9, 10).

Phytoplasmas reprogram their host plants’ metabolic, defense, and reproductive pathways *via* the production of small effector proteins, also called virulence factors. The majority of phytoplasma-induced symptoms are due to the secretion of these effectors by the bacteria during infection and result in changes to host plant regulatory and developmental networks. To date, three different classes of effector proteins have been identified. SAP11_AYWB_, first identified in Aster Yellows Witches’ Broom, directly binds and destabilizes host plant teosinte branched1-cycloidea–proliferating cell factor transcription factors resulting in the witches’ broom phenotype (11). The second class are small peptides, including TENGU, which affect phytohormone levels by downregulating auxin responsive genes, resulting in dwarfism, sterility, and witches’ broom (12, 13, 14). A third class of effectors has been shown to be highly conserved based on available genome sequencing data of different phytoplasma strains (15, 16). This class comprises a family of small, approximately 10 kDa proteins and induces the phyllody phenotype, an abnormal conversion of floral organs into leaf-like structures. These potent effectors have been named “phyllogens” for phyllody-inducing genes. The encoded small proteins, referred to collectively as phyllogens, include SAP54 from Aster Yellows Witches’ Broom and PHYL_OY_ from *Ca. P. asteris* onion yellows (OYs) (3, 4, 17, 18, 19).

Plant phyllody is one of the most recognizable signs of phytoplasma infection and one of the most economically costly as it deleteriously affects flower, seed, and fruit production, rendering the host plant unable to reproduce and creating what has been termed a «zombie» - a dead-end organism existing only to propagate the infecting phytoplasma (4, 20). The molecular mechanisms underlying the phyllody phenotype are due to the interactions of phyllogens with host plant MADS transcription factors (MTFs), which ultimately results in MTF degradation by the 26S proteasome (5, 19). While phytoplasma are phloem-localized, the effector peptides and small proteins they secrete are transported across the phloem companion cells, and, in the cases of phyllogens, to the shoot apical meristem where they bind a subset of MTFs critical for flowering, floral meristem identity, and floral organ morphogenesis. The MTFs, APETALA1 (AP1) and cauliflower (perianth organ development and floral meristem identity) and the SEPALLATAs (SEPs) (organ development and determinacy), have been identified as primary targets of phyllogens in Arabidopsis and other plant species (3, 5, 18, 19, 21). Of note, while MTFs are present in all eukaryotes, including insects, only a subset of plant MTFs possesses the requisite domains for interaction with phyllogens.

Members of the plant-specific “MIKC” or type II MADS family are the interaction targets of phyllogens. The MIKC MTFs have a modular and structurally well-defined four-domain organization (22). These domains consist of a dimeric DNA-binding MADS domain (**M)** and a ∼30 amino acid alpha helical intervening domain (**I**) critical for dimerization specificity, which are conserved in all eukaryotes (23, 24). The MI domains are followed by a coiled-coil keratin-like tetramerization domain (**K**) and a largely unstructured and sequence-variable C-terminal domain (**C**). The tetramerization K domain is plant specific and provides the protein-binding interface for phyllogens (17, 21, 25). Interestingly, while angiosperms possess tens of MIKC-type MTFs, (*i.e.* Arabidopsis encodes over 50 MIKC-type MTFs), only a few MIKC MTFs involved in plant reproduction have been shown to interact with phyllogens. These phyllogen-targeted MTFs belong to the *AP1/SEP/*AGAMOUS-LIKE6 (*AGL6)* MADS superclade, and from Arabidopsis include AP1, CAL, SEPs, and AGL6, a floral activator (3, 5, 18, 19). Protein–protein interactions have been mapped to the K domain for AP1 and SEPs, which bears structural similarity to the phyllogen coiled-coil. The molecular basis of interaction specificity with only a few floral-specific MTFs is still unclear.

Current data suggest that phyllogens are highly specific to only a few MTFs critical for flower morphogenesis and use these transcription factors to reprogram reproductive development in the host plant. Here, we examine the molecular and atomic level determinants of PHYL_OY_–MTF interactions, present structural data on PHYL_OY_–MTF complexes, demonstrate the effect of PHYL_OY_ on MTF DNA-binding and show, *in planta*, using the *AP1* and *SEP3* promoters that *PHYL*_*OY*_ expression may be targeted to specific whorls, with *PHYL*_*OY*_/MTF interaction patterns strongly correlating with the observed floral phenotypes.

## Results

### PHYL_OY_ interacts with a subset of MTFs *via* the K domain

**Figure 1:**
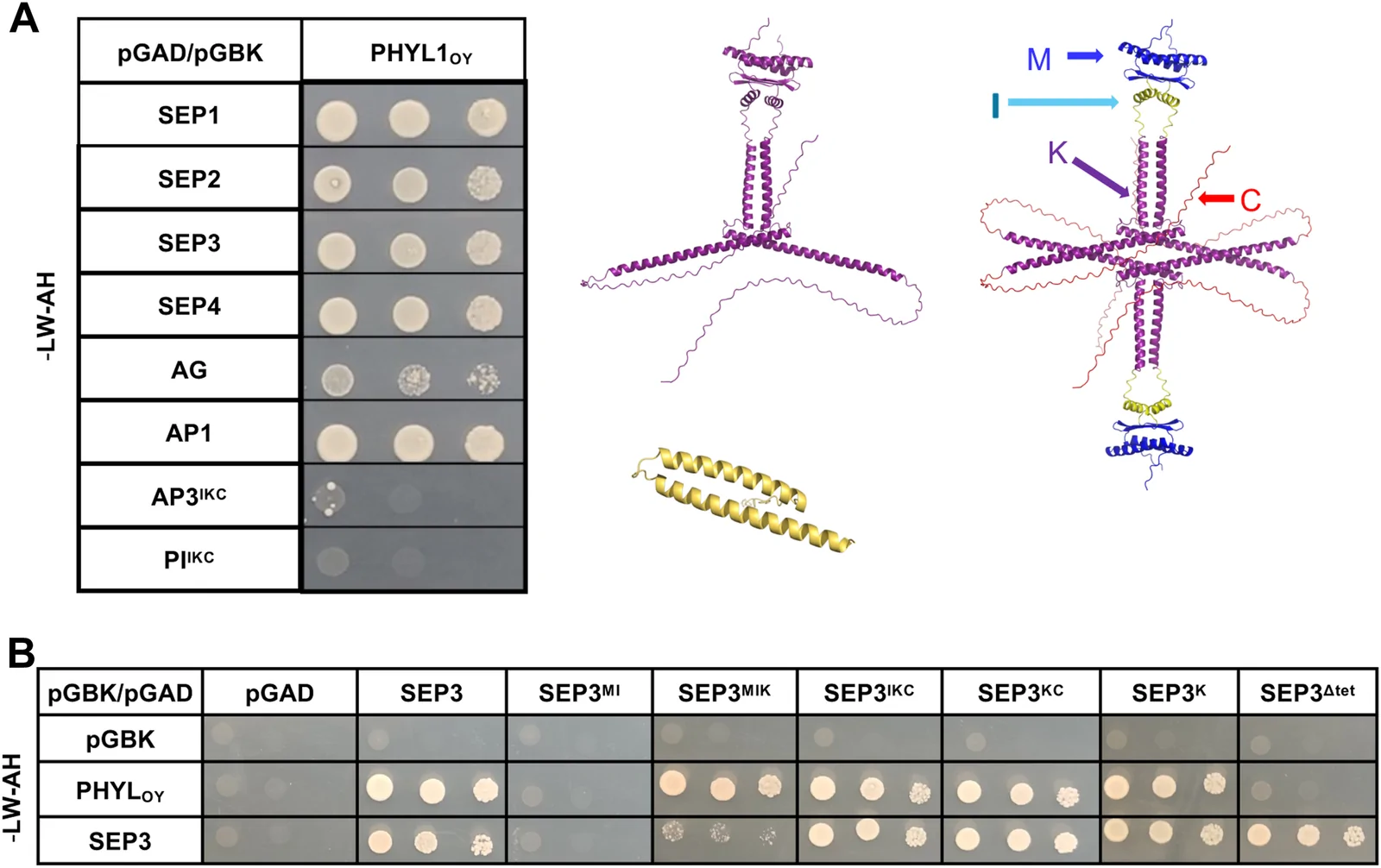
**Interactions between MADS transcription factors and PHYL**_**OY.**_*A,* at *left*, SEP1, SEP2, SEP3, SEP4, AG, and AP1 interact with PHYL_OY_ as demonstrated by yeast two-hybrid. No interactions were detected between PHYL_OY_ and AP3^IKC^ or PI^IKC^ constructs. The M domains of AP3 and PI were deleted as the full-length proteins behave poorly and exhibit no interactions in yeast two-hybrid (Fig. S1). At *right*, from *top left* clockwise cartoon structures generated using AlphaFold (AF3) of the SEP3 dimer in *purple*, the SEP3 homotetramer with the M, I, K, and C domains labeled and colored *blue*, *yellow*, *purple,* and *red*, respectively, and the experimental structure (PDB: 6JQA) of the PHYL_OY_ monomer in *yellow*. The MTFs and PHYL_OY_ have alpha helices that interact *via* coiled-coil formation. *B,* domain interactions between SEP3 and PHYL_OY_ were tested. MI, MADS and intervening domains, residues 1 to 90; MIK, MADS, intervening, and keratin-like domains, residues 1 to 173; IKC, intervening, keratin-like, and C-terminal domains, residues 59 to 252; KC, residues 91 to 252; K, residues 75 to 178, and Δ tet = deletion of residues 160 to 174 in the K domain. C domain, C-terminal domain; M domain, MADS domain; MTF, MADS transcription factor; PHYL_OY_, onion yellows phyllogen; SEP, SEPALLATA.

Previous studies demonstrated interactions between phyllogens and MTFs from the *AP1/SEP/AGL6* superclade (5, 18, 19). Using PHYL_OY_, we confirmed the interaction between PHYL_OY_ and SEP1, SEP2, SEP3, SEP4, and AP1 from *Arabidopsis thaliana* by yeast two-hybrid, as previously reported (18, 19). In addition, we also observed no interaction with AP3 and PISTILLATA (PI) and a weak but detectable interaction with AG, which was not previously reported (Figs. 1*A*, S1, and S2). Using SEP3 as a representative MTF, we further mapped the interaction of PHYL_OY_ to the K domain of SEP3, as previously reported in the literature (18) (Fig. 1*B*). Interaction with PHYL_OY_ was abrogated in a SEP3 splice variant carrying a deletion from amino acid 160 to 174 in the second helix of the K domain corresponding to the MADS tetrameric interface (Fig. 1*B*). To further elucidate the molecular determinants for these interactions and to determine the stoichiometry of the PHYL_OY_–MTF complexes, we performed a series of *in vitro* experiments using the K domains of homomeric SEP3 and AG complexes and the heteromeric SEP3–AG complex.

Size-exclusion chromatography multiangle light scattering (SEC-MALS) was performed on different PHYL_OY_–MTF complexes (Fig. S3). As MTFs form dimers and tetramers *via* their K domains and this domain has been demonstrated to provide the interface for phyllogen–MTF interactions, we recombinantly overexpressed and purified PHYL_OY_ and the K domains of SEP3^75-178^, AG^90-189^, and the heteromeric SEP3^75-178^/AG^90-189^ complex. PHYL_OY_ and the MTFs were purified separately and then mixed with an excess of PHYL_OY_ and the resulting complexes characterized by SEC-MALS. AG^90-189^ when expressed alone exhibited poor stability and we were not able to confirm the interaction with PHYL_OY_. However, SEP3^75-178^ and the SEP3^75-178^/AG^90-189^ hetero-oligomer exhibited complex formation with PHYL_OY_ and eluted from size exclusion at the same retention volume, suggesting the proteins form species with the same stoichiometry. SEC-MALS confirmed the stoichiometry of the SEP3^75-178^/PHYL_OY_ and SEP3^75-178^/AG^90-189^/PHYL_OY_ complexes as 2:1 for SEP3^75-178^: PHYL_OY_ and 1:1:1 for SEP3^75-178^/AG^90-189^/PHYL_OY_. These experiments demonstrate that PHYL_OY_ binds the dimeric form of the K domains of MTFs and PHYL_OY_ binding is not compatible with the tetrameric form of MTFs. To determine the effect of PHYL_OY_ on DNA binding of the full-length MTFs, EMSAs were performed.

### PHYL_OY_ alters MTF DNA-binding patterns *in vitro*

**Figure 2:**
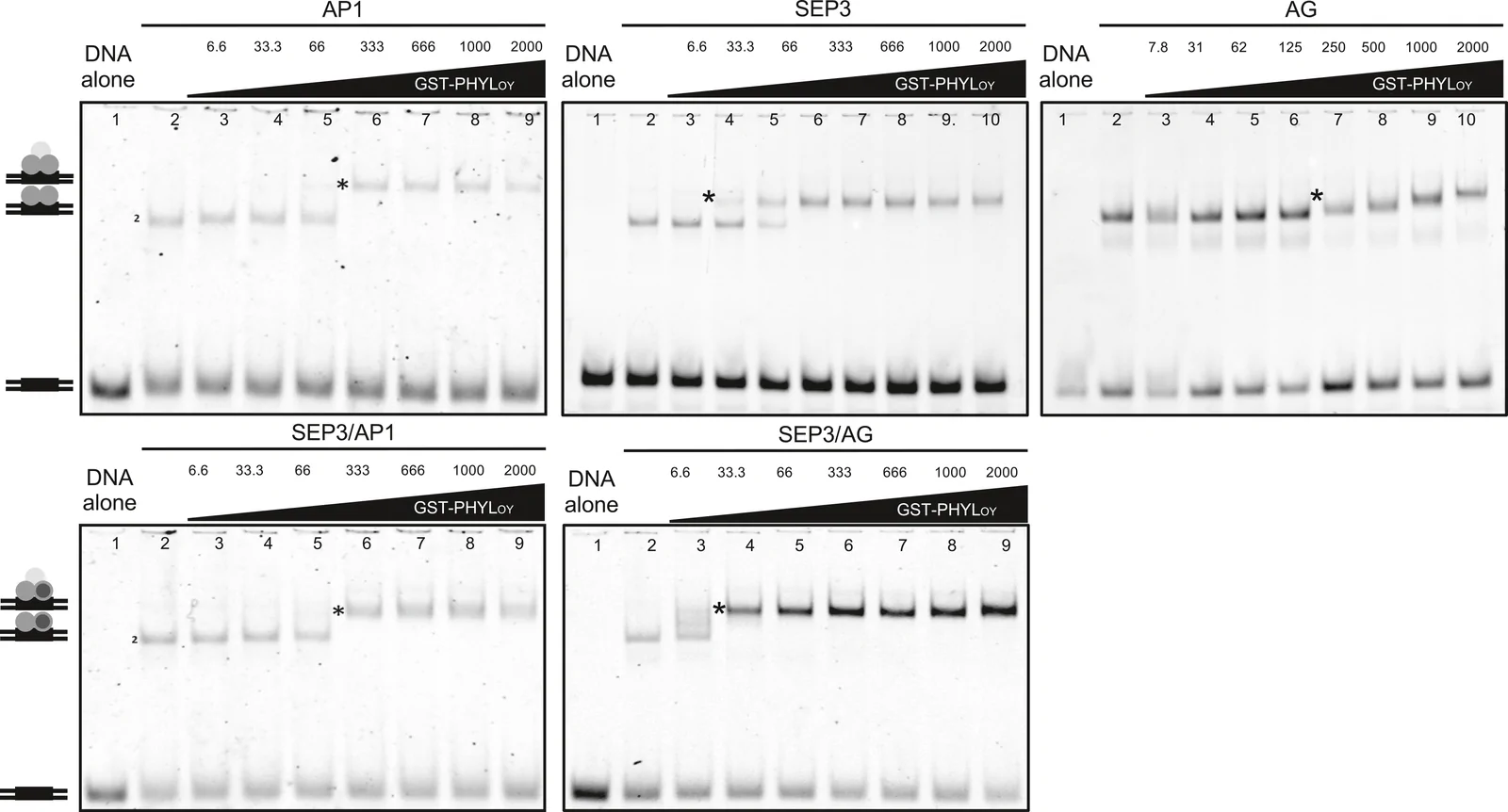
**EMSAs for MTFs and GST–PHYL**_**OY.**_ MTFs were produced by *in vitro* transcription translation and incubated with a fluorescently labeled DNA probe from the *SEP3* promoter containing a single CArG box MTF-binding site. Purified GST-PHYL_OY_ produced from bacterial expression was titrated from *low* to *high* concentrations as noted for each lane with concentrations given in nanomolar. The *asterisk* denotes formation of an MTF/GST–PHYL_OY_ complex. AG, AGAMOUS; AP1, APETALA1; SEP3, SEPALLATA3. Complexes are depicted schematically at *left* of the gels. MTF, MADS transcription factor; PHYL_OY_, onion yellows phyllogen; SEP, SEPALLATA.

EMSA experiments with different combinations of MTFs and PHYL_OY_ were carried out to examine *in vitro* DNA binding of the MTFs in the presence of PHYL_OY_. We used a DNA probe with a single CArG box (CCA_6_GG) MTF-binding site and GST-tagged PHYL_OY_ protein to more easily differentiate the complexes formed and to discriminate between MTF and PHYL_OY_–MTF complexes. The EMSA experiments (Fig. 2) were performed with 10 nM Cy5-labeled DNA and a constant concentration of MTFs produced using *in vitro* transcription–translation. PHYL_OY_ was recombinantly overexpressed, purified, and titrated from 0 nM to 2000 nM into the MTF-DNA binding mixture. The proteins and DNA were allowed to incubated for ∼60 min prior to loading onto the gel. EMSAs demonstrated a clear supershift for AP1/GST-PHYL_OY_, SEP3/GST-PHYL_OY_, AG/GST-PHYL_OY,_ AP1/SEP3/GST-PHYL_OY_, and SEP3/AG/GST-PHYL_OY_. Interestingly, AG is not a member of the AP1/SEP/AGL6 superclade and no interaction with phyllogens has been previously reported, to our knowledge. These results suggest that phyllogens may have a broader interaction specificity with MTFs. The highest affinity binding under the conditions tested was with SEP3/AG heterodimers, which exhibited a clear supershift at <30 nM PHYL_OY_. To better understand the amino acids required for PHYL_OY_ binding to MTFs, we probed the putative binding interface *via* site-directed mutagenesis and structural studies.

### Hydrophobic residues at the MTF tetramerization interface are required and sufficient for phyllogen interaction

**Figure 3:**
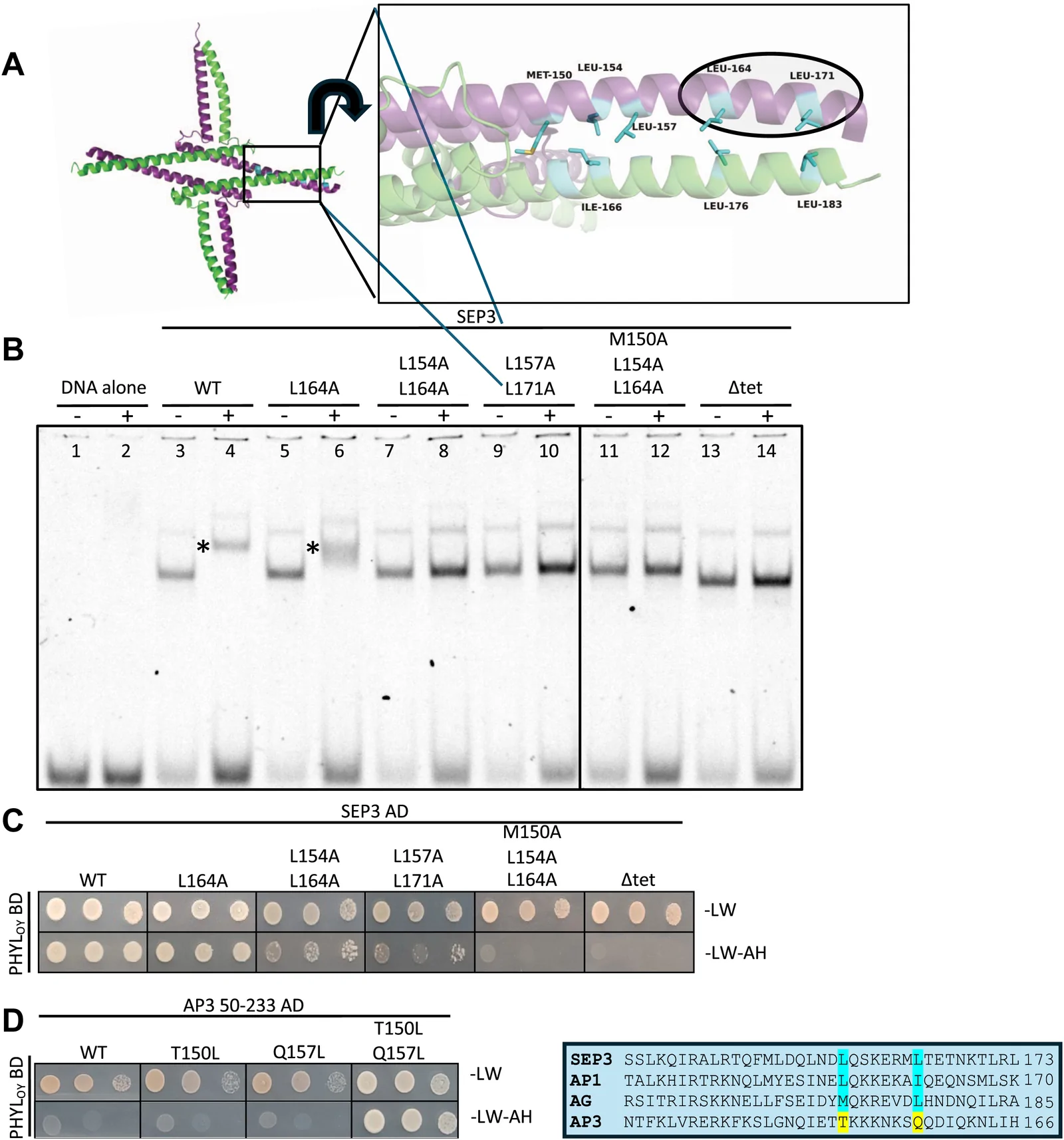
**Identification of key residues for interactions between MTFs and PHYL**_**OY.**_*A, cartoon* of SEP3 (*purple*) and AG (*green*) heterotetramer structure (PDB: 8CRA) with a zoom of hydrophobic residues located at the tetramerization interface. Zoom cartoon is in partial transparency for clarity. Residues are depicted as *sticks* and colored by atom with carbons in *cyan*. The *circled region* corresponds to residues 160 to 174. *B,* EMSA of SEP3 WT and point mutants in the absence (-) and presence (+) of 2 mM GST-PHYL_OY_. *Asterisks* denote SEP3/GST-PHYL_OY_ complex formation. DNA probe as per Figure 2. *C,* yeast two-hybrid demonstrating interactions between SEP3, with an increasing number of point mutations resulting in less detectable interactions with PHYL_OY_. Δtet refers to a deletion of residues 160 to 174 for SEP3. *D,* gain-of-function experiment in which mutating residues in AP3 (IKC domains) to the corresponding SEP3 residue resulted in interactions between AP3 IKC and PHYL_OY_ based on yeast two-hybrid (*left*). At *right*, partial sequence alignment with mutated residues highlighted in *yellow*. Numbering as per AP3. AP3, APETALA3, PHYL_OY_, onion yellows phyllogen; SEP, SEPALLATA.

Recent studies have reported the importance of the hydrophobic residues and more particularly leucines in the second helix of the K domain for the interaction between phyllogens and SEP3 (5, 18, 19). To further investigate where PHYL_OY_ binds to the MTFs and which amino acids are required for this interaction, we mutated the hydrophobic residues of SEP3 tetramerization interface (Figs. 3 and S4). The single mutation SEP3^L164A^ resulted in only minimal disruption of the MTF–PHYL_OY_ complex based on EMSA and yeast two-hybrid (Fig. 3*B*, lane 6 and *C*). In contrast, double mutations (L154A/L164A and L157A/L171A, Fig. 3*B*, lanes 8 and 10) resulted in no complex formation with PHYL_OY_ and strongly attenuated interactions in yeast (Fig. 3*C*). Mutating three sites (M150A, L154A, L164A, Fig. 3, lane 12) abrogated interactions in EMSA and yeast two-hybrid assays. A splice variant of SEP3 formed by exon skipping that deletes amino acids 160 to 174 in the K domain also completely abrogated interactions with PHYL_OY_ based on EMSA experiments (Fig. 3*B* lane 14).

In parallel to the loss of interaction studies, we sought to engineer gain of interactions between the MTF, AP3, and PHYL_OY._ AP3, a B-class MTF important for petal and stamen organ identity does not interact with PHYL_OY_ in yeast two-hybrid assays and lacks the hydrophobic residues present in SEPs, AG and AP1 at the K domain tetramerization interface. Based on sequence alignments of AP1, SEP3, and AP3, the single AP3 mutations, T150L and Q157L that corresponded to hydrophobic residues in SEP3, AG and AP1, and the double mutation, T150L/Q157L, were generated and tested for interaction with PHYL_OY_ by yeast two-hybrid assays (Figs. 3*D* and S5). AP3 WT and the single point mutations did not interact with PHYL_OY_; however the double mutation, AP3^T150L/Q157L^, resulted in a positive interaction with PHYL_OY_, recapitulating the positive interaction observed for PHYL_OY_ with SEPs, AG, and AP1. These results further support that hydrophobic amino acids at the tetramerization interface are critical for PHYL_OY_ binding and discriminatory for the observed interactions with MTFs.

### Structural studies of SEP3/SEP3/PHYL_OY_ and SEP3/AG/PHYL_OY_

**Figure 4:**
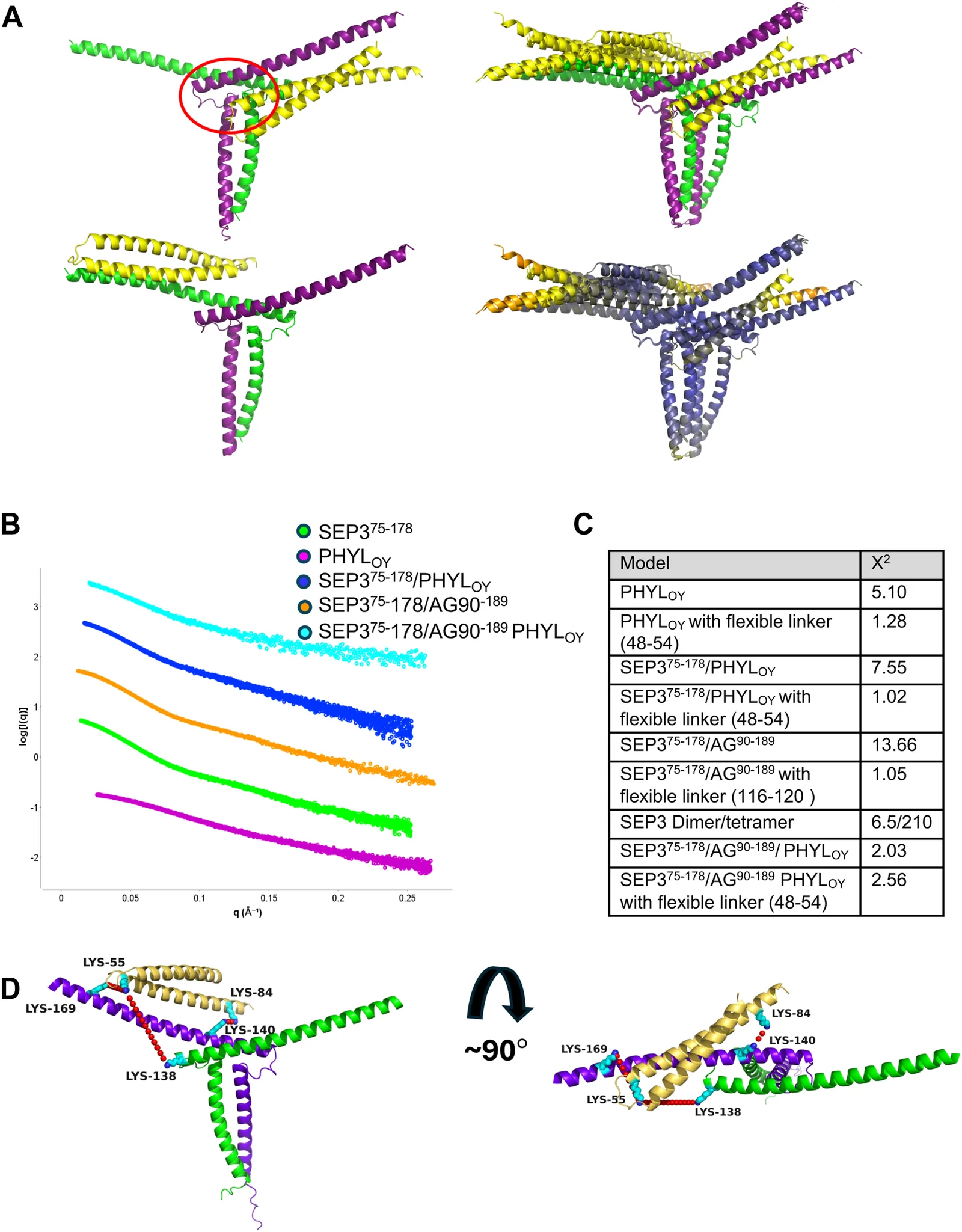
**Structural data for SEP3/AG/PHYL complex.***A,* AlphaFold (AF3) models for the SEP3/AG/PHYL_OY_ complex demonstrating variable positioning of the PHYL_OY_ protein along the tetramerization interface. Two models are shown at *left* and overlays are shown at *right* colored by chain (*top*) or pLDDT score (*bottom*). *B,* SAXS curves for the different complexes measured with curves offset for comparison. *C,* SAXS fitting statistics for the different complexes using a single model or a flexible ensemble due to predicted flexibility of the PHYL_OY_ or MTF protein from the linker region between helices, as shown *circled* in (*A*), *left*. The chi-square values improved with added flexibility for the majority of complexes. *D,* HADDOCK model with cross-linking mass spectrometry interactions shown between lysine residues, with lysines colored by atom with carbons in *cyan*. The mass spectrometry–based model was similar to the AF3 models. SEP3 is in *purple*, AG in *green,* and PHYL_OY_ in *yellow*. MTF, MADS transcription factor; PHYL_OY_, onion yellows phyllogen; SAXS, small-angle X-ray scattering; SEP, SEPALLATA.

In order to better characterize the MTF-PHYL_OY_ complexes at the atomic and molecular level and to help determine the most likely orientation of the PHYL_OY_ helices with respect to the MTF tetramerization interface, we performed a series of structural studies using small-angle X-ray scattering (SAXS) and cross-linking mass spectrometry (XL-MS). Structure prediction using AlphaFold3 (AF3) yielded different possible multimer conformations for the MTF–PHYL complexes (Fig. 4*A*) (26). As the interface predicted template modeling scores were very similar, we were not able to determine which structure was most likely to represent the physiological complex. In addition, previous studies using XL-MS proposed different putative conformations of SEP3 with the related phyllogen, PHYL_PnWB_ (21). To address this ambiguity, we recombinantly overexpressed and purified the complexes SEP3^75-178^/PHYL_OY_ and SEP3^75-178^/AG^90-189^/PHYL_OY_.

All complexes were subjected to crystallization trials; however, we did not obtain well-diffracting crystals of any of the MTF–PHYL_OY_ complexes. In order to obtain structural data, albeit at low resolution, we performed SAXS experiments on PHYL_OY,_ SEP3^75-178^, SEP3^75-178^/AG^90-189^, SEP3^75-178^/PHYL_OY_, and SEP3^75-178^/AG^90-189^/PHYL_OY_ (Fig. 4, *B* and *C*). SEC-SAXS was used to remove any aggregates from solution and to optimize data quality (27, 28). All samples eluted as single peaks with a stable *R*_*g*_ across the peak. SAXS data and statistics are given in Fig. S6. When observing the R_g_ of the different species, it is noticeable that SEP3^75-178^ (40.2 Å) is larger than SEP3^75-178^/AG^90-189^ (36.1 Å). The P(r) curves (Fig. S6*C*) also show SEP3^75-178^ D_max_ is the largest at 154 Å. This is consistent with SEP3^75-178^ forming a tetramer in solution. SEP3^75-178^/AG^90-189^ has a smaller D_max_ (112 Å) indicating a more compact structure, consistent with the SEC-MALS (Fig. S3) indicating a dimer or dimer/tetramer in solution. The addition of PHYL_OY_ to SEP3^75-178^ and SEP3^75-178^/AG^90-189^ resulted in species that give a smaller Rg and D_max_ (Fig. S6*A*). This would suggest a compaction or a decrease in flexibility in the complex form. The normalized Kratky plots (Fig. S6*B*) show that the complexes are all elongated, indicating flexibility in the structures.

To validate these SAXS data, fitting of the experimental scattering curves with the different models (experimental and AF3) was performed (26). The best fits yielded chi-square values ranging from 2 to 200 (Figs. 4 and S7). The worst chi-square values correspond to fitting a dimer to the SEP3^75-178^ data. Fitting of a tetramer resulted in a decrease in the chi-square to 6.48, suggesting that SEP3^75-178^ is primarily tetrameric in solution. To investigate whether accounting for flexibility due to linker regions between the folded domains of the MTFs could better explain the experimental data, an ensemble of three different conformations was used, resulting in improved chi-square values ranging from 1.02 to 1.28 for all complexes, except for the SEP3^75-178^/AG^90-189^/PHYL_OY_ complex that gave a higher chi-square value with added flexibility. This suggests that the complex is more constrained, favoring only 1 form. In contrast, the SEP3^75-178^/PHYL_OY_ complex shows a bimodal distribution in the ensemble fit, consistent with a bimodal distribution observed in the P(r) curve (Fig. S6*C*) and the complex sampling two states. Taken together, these experiments indicate that the most likely conformation of the complexes corresponds to PHYL_OY_ binding primarily to 1 MTF monomer (Fig. 4*A*, left), with one of the PHYL_OY_ helices exhibiting some flexibility (Figs. S6 and S7), likely due to this region of the protein only weakly interacting with the MTF dimer.

### Cross-linking mass spectrometry

To determine the orientation of PHYL_OY_ with respect to the MTF dimer and to better understand the high-affinity binding to the SEP3^75-178^/AG^90-189^ heterodimer, XL-MS were performed on SEP3^75-178^/AG^90-189^/PHYL_OY_. Previous studies of SEP3 K domain with phyllogen from peanut witches’-broom phytoplasma using XL-MS and an amine reactive crosslinker, bis(sulfosuccinimidyl) suberate (BS3), suggested the proteins form a quadruple stranded coiled-coil (21). We initially tested three different crosslinkers: BS3, disuccinimidyl dibutyric urea (DSBU) and 4-(4,6-dimethoxy-1,3,5-triazin-2-yl)-4-methylmorpholinium chloride. As DSBU gave a complete cross-linking reaction on both complexes without inducing aggregation, we performed the mass spectrometry acquisition on these samples (Fig. S8*A*). A total of ∼30 to 35 crosslinked peptides per replicate were identified, with a high degree of reproducibility (Fig. S8*B*). The results showed a total of three crosslinked lysine pairs reproducibly detected between SEP3 and PHYL_OY_ (SEP3^K54^/PHYL_OY_^K86^, SEP3^K68^/PHYL_OY_^K86^, and SEP3^K97^/PHYL_OY_^K57^) and two pairs between AG and PHYL_OY_ (AG^K99^/PHYL_OY_^K99^ and AG^K51^/PHYL_OY_^K86^) (Table S1 and Fig. 4*D*). Crosslinked sites provided distance constraints between ∼7 and ∼31 Å (considering the flexibility of lysine side-chains, the α-carbon backbone, and the length of the cross-linking reagent) with an average cut-off distance of ∼33 to 35 Å (29, 30). These interprotein crosslinks were used as distance restraints for *in silico* docking of PHYL_OY_ to the SEP3-AG dimer with HADDOCK (31, 32). The HADDOCK results were highly similar to the AF3 derived model and consistent with the SEC–SAXS data (Fig. 4).

### *In vivo* activity of *PHYL*_*OY*_ using whorl-specific promoters

We expressed *PHYL*_*OY*_ under the control of the *35S*, *AP1*, and *SEP3* promoters in order to characterize the *in vivo* activity of PHYL_OY_ and its effect on whorl-specific MTF complexes. Based on our *in vitro* experiments and yeast two-hybrid assays, PHYL_OY_ preferentially interacts with the SEP3-AG heterodimer and we wondered whether this would result in increased fourth whorl developmental defects, as fourth whorl organ identity and determinacy is dependent on SEP-AG activity (33). As shown in previous studies and reproduced here, the *35S* constitutive promoter resulted in defects throughout the flower, with determinacy and fourth whorl organ development the most affected (Fig. S9) (18). In contrast, expressing *PHYL*_*OY*_ under control of the *AP1* or *SEP3* promoter resulted in whorl-specific phenotypes, suggesting the *PHYL*_*OY*_ is not efficiently transported to different tissues.

**Figure 5:**
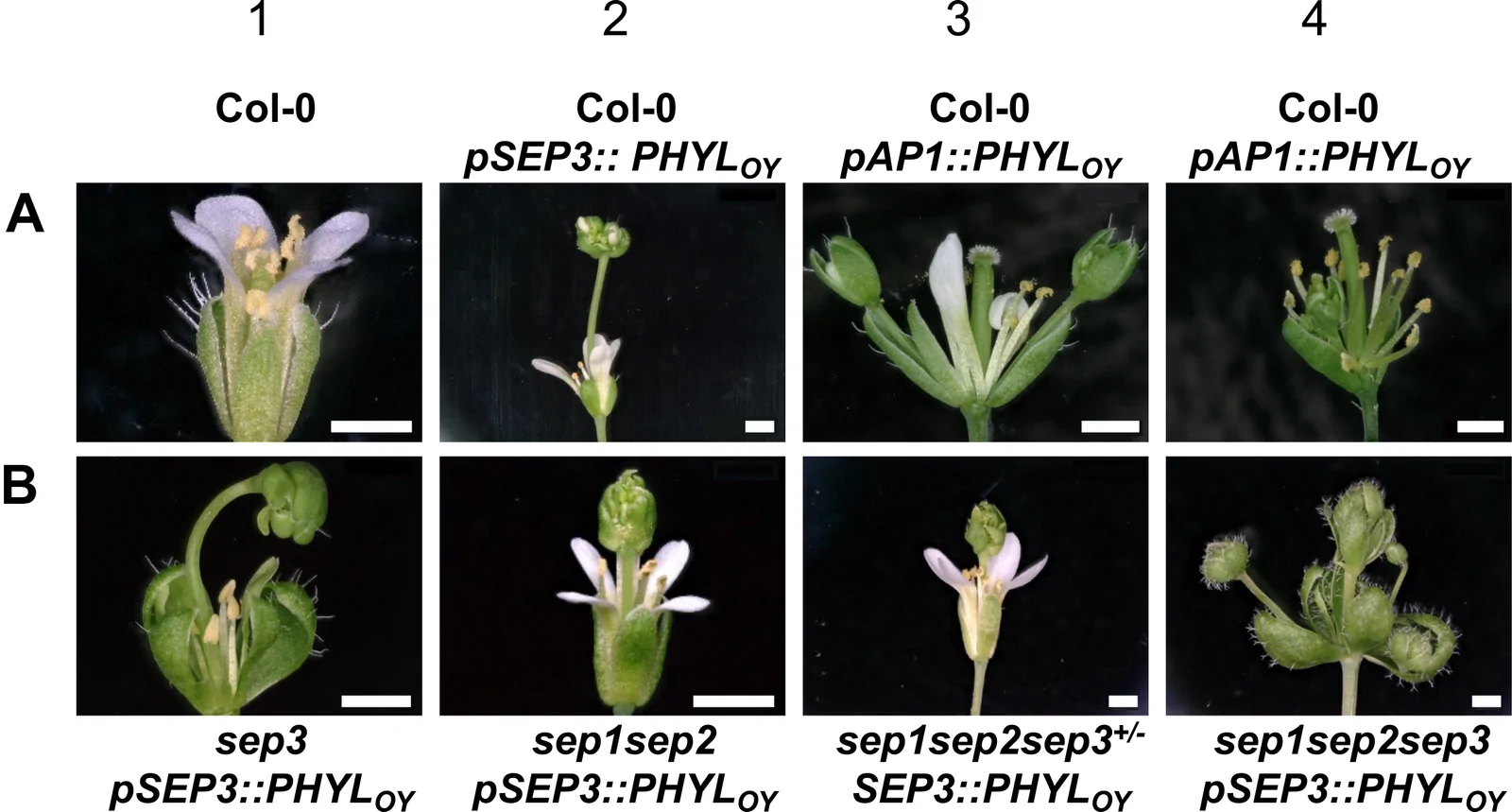
**Phenotypes of Arabidopsis plants expressing *PHLY1***_***OY*.**_*A,* Col-0 (WT), Col-0 transformed with *pSEP3::PHYL1*_*OY*_ and *pAP1::PHYL1*_*OY*_. Only 1 plant out of 20 expressing *PHYL1*_*OY*_ under the *SEP3* promoter exhibited indeterminacy (A-2) likely due to *SEP* redundancy. 19/20 plants resembled WT. The majority of the *pAP1::PHYL1*_*OY*_ plants, however, exhibited *PHYL1*_*OY*_-related phenotypes with 6/13 plants showing fewer petals in the second whorl and some secondary buds (A-3) and 4/13 plants exhibiting stronger homeotic conversion of first whorl organs into bracts, a complete lack of petals in the second whorl and secondary buds, recapitulating the *ap1* loss-of-function phenotype (A-4). *B,* different *sep* mutant combinations expressing *pSEP3::PHYL1*_*OY*_ show enhanced defects as compared to WT plants expressing *pSEP3::PHYL1*_*OY*_. The majority of *sep3* plants expressing *pSEP3::PHYL1*_*OY*_ (17/20) showed delayed flower opening and an indeterminacy phenotype in the fourth whorl for 3/20 (B-1). The majority of the double *sep1 sep2* (13/14) and the triple *sep1 sep2 sep3*^*+/−*^ (14/14) plants showed strong indeterminacy phenotypes in the fourth whorl (B-2 and B-3). 4/4 triple mutant *sep1 sep2 sep3 plants* expressing *pSEP3::PHYL1*_*OY*_ recapitulated the *sep1 sep2 sep3 sep4* quadruple mutant phenotype, with indeterminacy and the conversion of all floral organs to leaf-like structures. The scale bar represents 1 mm. PHYL_OY_, onion yellows phyllogen; SEP, SEPALLATA.

WT Col-0 plants expressing *pAP1:: PHYL*_*OY*_ were primarily affected in the first and second whorl, where *AP1* expression is highest. The strongest mutant phenotypes, corresponding to approximately 13% of primary transformants phenocopied the *ap1* loss-of-function mutant (34) (Figs. 5 and S9). Sepals and petals were absent and first and second whorl organs were replaced by bracts. New floral buds consisting of stamen and carpels formed in the axil of the transformed sepals with no petals present (∼20%).

For Col-0 expressing *pSEP3:: PHYL*_*OY,*_ ∼50% of the primary transformants did not show a noticeable phenotype and 30% only exhibited a delay in flower opening with all organs present and development as in the WT. Approximately 8% of the plants exhibited normal development in the first, second, and third whorls with only the fourth whorl affected with a low seed production and poorly developed ovules. The strongest phenotypes showed bracts in the first whorl, immature organs in the second and third whorl, and carpeloid structures completely lacking seeds (Fig. 5).

As *A. thaliana* has four *SEPALLATA* genes (*SEP1-4*) with overlapping and (semi-)redundant functions, we also transformed different *sep* mutants with *pSEP3::PHYL*_*OY*_ to investigate whether the phenotypes would be stronger in a background with reduced SEP protein expression. Fig. S9 shows the phenotypes of the *sep3*, *sep1 sep2, sep1 sep2 sep3,* and *sep1 sep2 sep3*^+/−^ mutant backgrounds. s*ep1 sep2* plants exhibit WT-like floral development and a single copy of *SEP3* is sufficient to trigger all floral organ identity programs (35). These mutants were transformed with *pSEP3*::*PHYL*_*OY*_. As shown in Figure 5, reducing the amount SEP alleles present resulted in stronger phenotypes in plants expressing *PHYL*_*OY*_, with the fourth whorl the most susceptible to defects. In the most affected mutants, no seeds were produced although organ development in whorls 1 to 3 was normal.

## Discussion

Mimicry of host plant protein–protein interaction surfaces is a crucial mechanism phytoplasmas use to hijack plant development. PHYL_OY_ is an elegant example, targeting the tetramerization interface of a subset of MTFs critical for flower morphogenesis. Based on phylogenetic reconstructions, *AP1* and *SEP3* are closely related MADS genes in flower development and are members of the *AP1/SEP/AGL6* MADS superclade (36). EMSA experiments demonstrate that PHYL_OY_ binds not only homomeric (SEP3 and AP1) and heteromeric (AP1-SEP3, SEP3-AG) MTF complexes but also directly interacts with AG, a member of a different MADS clade, expanding the known repertoire of MADS proteins PHYL_OY_ is able to directly bind (Fig. 2). In addition, while PHYL_OY_ is able to interact with the homomeric MTFs, the highest affinity in EMSAs was observed for the heteromeric SEP3–AG complex, suggesting residues from both MADS proteins may contribute to interactions with PHYL_OY_ (Fig. 2). These results were consistent with SAXS and XL-MS experiments that indicated interactions between the K domains of both SEP3 and AG with PHYL_OY_ (Fig. 4).

Mutagenesis targeting the MTF–PHYL_OY_ interface identified here and in previous studies (25, 37) demonstrate that hydrophobic leucine and methionine residues donated by the MTFs are critical for the interaction, with mutations of at least two hydrophobic residues resulting in abrogated interaction between SEP3 and phyllogen in yeast two-hybrid assays (Fig. 3*D*). These results are further supported by the complementary experiment using AP3, an MTF that does not interact with PHYL_OY_ in yeast two-hybrid and lacks some of these critical hydrophobic residues. Based on sequence alignments, two positions in AP3 were targeted for mutagenesis, Thr150 and Gln157, which correspond to two leucine residues in SEP3, leucine and isoleucine in AP1 and methionine and leucine in AG. The double AP3 mutant, AP3^T150L/Q157L^, strongly interacted with PHYL_OY_, whereas the WT and single mutants did not (Fig. 3). Patterns of hydrophobic residues thus likely act as “anchors” for the phytoplasma effector.

These *in vitro* results, with the SEP3-AG heterodimer exhibiting the strongest interaction with PHYL_OY_, are recapitulated *in planta*. Using different promoters—*35S*, *pAP1* and *pSEP3*—determinacy of the floral meristem and the identity of the floral organs was differentially affected (Figs. 5 and S9). The *35S* promoter, as previously reported and shown here, affects multiple whorls and, in the most severe cases, results in bracts and sepaloid organs in all whorls (18). Of note, determinacy and fourth whorl organ development, which require the SEP3–AG MTF complex, were most affected in plants transformed with the *35S:: PHYL*_*OY*_ construct. Expressing *PHYL*_*OY*_ under control of the *AP1* promoter resulted in defects in the first and second whorl, with the strongest phenotypes resembling the *ap1* loss of function mutant (34). No effects were observed in the third or fourth whorl and the plants expressing *PHYL*_*OY*_ under control of the *AP1* promoter all produced viable seeds, confirming that stamen and carpel development was not affected.

The *SEP* subgroup of MADS genes consists of *SEP1, SEP2, SEP3,* and *SEP4* in Arabidopsis and these genes have overlapping expression patterns and exhibit a high degree of functional redundancy. Single *SEP* mutants have no observable phenotype and only in the triple *sep1 sep2 sep3* and quadruple *sep1 sep2 sep3 sep4* mutant are floral organs converted to sepals or leaves, respectively (38, 39). WT plants expressing *PHYL*_*OY*_ under the *SEP3* promoter were relatively mildly affected, with the majority of transformants resembling WT and ˂10% of plants exhibiting defects in the fourth whorl. Titrating the amount of *SEP* activity *via* single, double, and triple *SEP* mutants (*sep3*, *sep1 sep2*, *sep1 sep2 sep3*^*+/−*^), however, resulted in stronger phenotypes in mutant plants expressing *pSEP3:*:*PHYL*_*OY*_ (Fig. 5). In all cases, the fourth whorl was most affected with few or no seeds produced and, in severe cases, homeotic conversion of carpels to sepaloid structures was observed. In contrast, whorls 1, 2, and 3 retained normal floral organs even though organ development in these whorls requires SEP-containing MTF tetramers. These results may be reflective of the preference of PHYL_OY_ to bind to fourth whorl organ identity complexes containing the SEP–AG complex and even low concentrations of PHYL_OY_ would be sufficient to disrupt the function of these MTFs. Of note, by using different promoters, we were able to target specific MTF complexes and differentially affect floral organ development. This suggests PHYL_OY_ may be a useful tool to probe specific functions of AP1- and SEP-containing MTFs in a stage-, tissue- or organ-specific manner throughout flower development.

Phyllogens have coevolved with the MTFs to target the MADS tetramerization interface and use this as a docking surface (20). All physiological angiosperm MTF tetramers that interact with phyllogens have at least 1 copy of a AP1/SEP/AGL6 superclade protein that act as tetramerization hubs and provide an anchor interface for phyllogen binding. Interestingly, gymnosperms do not have *AP1* or *SEP* genes, but their likely physiological MTF complexes in reproductive development do contain an *AG* class gene and are able to form tetrameric complexes (40, 41). Yeast two-hybrid experiments demonstrated interactions between gymnosperm and fern MTFs and phyllogens (3), and our results here showing interactions between AG and PHYL_OY_ further supporting the key role of the tetramerization interface and specific hydrophobic residues as the required interaction surface for phyllogens. These findings suggest that the hydrophobic MTF tetramerization interface is an evolutionarily conserved protein–protein interaction surface present in gymnosperms and angiosperms that has been exploited by phyllogens during plant evolution.

## Experimental procedures

### Plant material and growth conditions

All experiments were performed using *A. thaliana* WT and *sep* mutants in the Col-0 background. The *sep3* and *sep1 sep2 sep3*^±^ mutants were used as previously described. Seedlings were grown in controlled growth chambers in long day conditions (16 h light**/**8 h dark) at 22 ^°^C for plant transformation and phenotype analysis. For the phenotype analysis, 35S::*PHYL*_*OY,*_ *pAP1::PHYL*_*OY*_*,* and *pSEP3::PHYL*_*OY*_ were used (33) and transformed into the appropriate background. The vector backbone, *pFP100*, allows GFP expression in seeds for selection of transformants and was used for all experiments (42).

### Plant transformation and floral phenotype analysis

For the generation of *sep3*, *sep1 sep2,* and *sep1 sep2 sep3* expressing p*SEP3:: PHYL*_*OY*_*, sep3, sep1 sep2,* or heterozygous *sep1 sep2 sep3*^*+/−*^ plants were transformed with p*SEP3:: PHYL*_*OY*_ using the floral dip method (43). All other transformations were performed in the WT Col-0 background. Transformants were selected based on the fluorescence of GFP-positive seeds.

Floral phenotypic analyses were performed by light microscopy on flower numbers 10–19 based on their order of emergence on T1 plants.

### Protein expression and purification

SEP3^(75-178)^ was cloned in *pESPRIT2* (44, 45) as previously described (46). SEP3^(75-178)^ and AG^(90-189)^ were cloned into the coexpression vector *pETDuet-1* (Novagen, Merck Millipore) as previously described (35). To obtain tagged AG^(90-189)^ alone, the SEP3^(75-178)^ sequence was removed by site-directed mutagenesis. *PHYL*_*OY*_ was cloned into *pGEX-4T-1* as previously described (17).

The MTF K domains and PHYL_OY_ proteins were overexpressed in *Escherichia coli ROSETTA2 (DE3)*. Cells were grown at 37 °C at 130 rpm on Luria-Bertani medium in the presence of chloramphenicol 34 μg mL^−1^ and kanamycin 50 μg mL^−1^ for *pESPRIT2* constructs or chloramphenicol 34 μg mL^−1^ and carbenicillin 100 μg mL^−1^ for *pETDuet-1* and *pGEX-4T-1* constructs. At an absorbance (A)_600nm_ of 0.6, the temperature was reduced to 17 °C and protein expression was induced with the addition of 0.5 mM IPTG for 16 h. Cells were pelleted at 4000 rpm for 30 min at 4 °C and resuspended in 50 mM Tris pH 7.5, 300 mM NaCl, and 1 mM Tris-(2-carboxyethyl)phosphine (TCEP). After sonication, the cell debris were pelleted at 12,000 rpm for 30 min at 4 °C and the supernatant was collected. The collected supernatant containing the soluble proteins was applied to a 2 ml nickel-nitrilotriacetic acid resin (Ni-NTA) column preequilibrated with 50 mM Tris pH 7.5, 300 mM NaCl, and 1 mM TCEP for the MTF proteins. After 1 h of incubation at 4 °C, the proteins bound were washed with 50 mM Tris pH 7.5, 300 mM NaCl, 1 mM TCEP, and 10 mM imidazole and then eluted with 50 mM Tris pH 7.5, 300 mM NaCl, 1 mM TCEP, and 250 mM imidazole. Elution fractions containing the protein of interest were pooled and dialyzed against 50 mM Tris, pH 7.5, 300 mM NaCl, and 1 mM TCEP in the presence of TEV protease overnight at 4 °C to cleave the tags. After removal of the TEV protease and uncut protein over a preequilibrated Ni-NTA column, the cleaved protein was concentrated to ∼8 mg/ml and applied to a size-exclusion fast protein liquid chromatography (FPLC) column (Superdex Increase 200 10/300 GL; GE Healthcare) preequilibrated with 50 mM Tris, pH 7.5, 300 mM NaCl and 1 mM TCEP. For the 6-His-MBP-(TEV)-AG^(90-189)^-S-tag purification, no TEV was added during the dialysis to keep the tags as cleavage resulted in precipitation of the AG construct.

For PHYL_OY_ the collected supernatant containing the soluble proteins was applied to a 2 ml GST-Sepharose column preequilibrated with 50 mM Tris pH 7.5, 300 mM NaCl, and 1 mM TCEP. After 1 h of incubation at 4 °C, the proteins bound were washed with 50 mM Tris pH 7.5, 300 mM NaCl, 1 mM TCEP, and then eluted with 50 mM Tris pH 7.5, 300 mM NaCl, 1 mM TCEP, and 20 mM reduced glutathione. Fractions containing the protein of interest were pooled and dialyzed against 50 mM Tris, pH 7.5, 300 mM NaCl, 1 mM TCEP, and 5 U thrombin per mg of PHYL_OY_ overnight at 4 °C. After the dialysis, the protein was concentrated to ∼8 mg/ml and applied to a size-exclusion FPLC column (Superdex Increase 200 10/300 GL; GE Healthcare) preequilibrated with 50 mM Tris, pH 7.5, 300 mM NaCl, and 1 mM TCEP.

### Yeast two-hybrid

The Matchmaker GAL4 Two-Hybrid System 3 (Clontech) was used as described for all yeast two-hybrid experiments (47). Briefly, *PHYL*_*OY*_ was cloned into the expression vector *pGBKT7* (Clontech) and the MTFs into the expression vector *pGADT7* (Clontech). The yeast strains Y187 and AH109 were transformed with the *pGBKT7* and p*GADT7* constructs, respectively. The *pGBKT7* and *pGADT7* transformed yeast were selected with solid medium lacking Trp (-W) and Leu (-L), respectively. After mating, the resulting transformants were selected with -LW medium. The selected strains were diluted at three different optical densities (A_600nm_ of 10, 1, and 0.1) onto two different selection plates containing -LW or -LWAH medium (lacking Leu, Trp, Ade, and His). The -LWAH medium was used to select diploid transformants presenting a positive protein interaction. Pictures were taken every day from day 1 to day 5.

### EMSA experiments

*AG* (At4g18960.1), *SEP3* WT (At1g24260.2), and *SEP3.3* (At1g24260.3) constructs in *pSP64* plasmid (Promega) were built as described previously (33). The *SEP3* L154A-L164A and L157A-L171A sequences were amplified from the yeast constructs and cloned into the *pSP64* plasmid. The *SEP3* L164A and M150A/L154A/L164A constructs were obtained by site-directed mutagenesis using the *SEP3* WT construct in *pSP64*. All *pSP64* constructs were used for *in vitro* transcription translation (Promega SP6 High Yield Expression System). EMSAs were performed as described (33) with 10 nM of *SEP3* promoter DNA containing one CArG box amplified by the primers 5′-GACGATAACTCCATCTTTCTATTTTG-3′ and 5′-CGTAATGGGAAGGGGACCTC-Cy5-3′. The binding buffer contained 7 mM Hepes pH 7.0, 1 mM BSA, 1 mM EDTA, 1 mM DTT, 2.5% Chaps, 6% glycerol, 0.06 mg/ml salmon sperm DNA, and 1.3 mM spermidine. Protein–DNA complexes were run on a nondenaturing 5% polyacrylamide gel for 90 min at 75V with a 1x 89 mM Tris-borate, 89 mM boric acid, 2 mM EDTA buffer.

### Size-exclusion chromatography multiangle light scattering

SEC-MALS experiments were conducted on a HPLC (Schimadzu) consisting of a degasser DGU-20AD, a LC-20AD pump, a autosampler SIL20-ACHT, a communication interface CBM-20A, a UV-visible detector SPD-M20A, a column oven XL-Therm (WynSep, Sainte Foy d’Aigrefeuille) and a static light scattering detector miniDawn Treos, a dynamic light scattering detector DynaPro NANOSTAR, a refractive index detector Optilab rEX (Wyatt, Santa-Barbara). 20 μl or 40 μl of samples (for SEP3^(75-178)^/PHYL_OY_ or SEP3^(75-178)^/AG^(90-189)^/PHYL_OY,_ respectively) were injected on a Superdex 75 10/300 GL, equilibrated at 4 °C, with the elution buffer containing 50 mM Tris pH 7.5, 300 mM NaCl, 1 mM TCEP, and at a flow rate of 0.5 ml/min.

### Small-angle X-ray scattering

SAXS data were collected at the bioSAXS beamline B21 at diamond light source, using a KW-402.5 size-exclusion column operated at 0.16 mL min^−1^ on an Agilent 1200 HPLC system, and at the BioSAXS beamline BM29 at the European Synchrotron Radiation Facility using an S75 column on the integrated Shimadzu HPLC system with a flow rate of 0.05 mL min^−1^ at room temperature (48, 49). The columns were equilibrated in 25 to 50 mM Tris pH 8.0, 300 mM NaCl, 1 mM TCEP.

Protein samples (50 μL at 1-6 mg mL^−1^ concentrations) of SEP3^(75-178)^, SEP3^(75-178)^/AG^(90-189)^, PHYL_OY_, SEP3^(75-178)^/PHYL_OY_, or SEP3^(75-178)^/AG^(90-189)^/PHYL_OY_ were loaded onto the size-exclusion column, and SAXS frames were recorded continuously with 1 to 3 s exposure times. Initial data handling was performed automatically using the Dahu pipeline, which generated radially integrated, calibrated, and normalized one-dimensional scattering profiles for each frame (50). Further processing was carried out in Scatter IV (27). Frames corresponding to the buffer baseline (50–100 frames) were selected, and sample elution frames were inspected using heatmap visualization. Sets of consistent sample frames (cyan clusters) were selected and merged. For each SEC run, merged frames corresponding to the highest protein concentration were combined to produce an idealized scattering curve, which was used for subsequent analysis and modelling.

Data reduction, buffer subtraction, averaging, Guinier analysis for determination of *R*_*g*_, and calculation of P(r) distributions were performed using ScÅtter (27). The forward model used for flexibility modeling and theoretical scattering profile fitting were carried out using FoxS (51, 52). Explicit solvent model fitting was performed with the WAXSiS server (https://academic.oup.com/nar/article/43/W1/W225/2467862?login=true).

### Cross linking mass spectrometry

The SEP3^(75-178)^_2_/AG^(90-189)^_2_ and SEP3^(75-178)^/AG^(90-189)^/PHYL_OY_ complexes were chemically crosslinked at 26 μM with 50 to 100 fold excess of DSBU (2 mM), BS3 (2 mM) and (4-(4,6-dimethoxy-1,3,5-triazine-2-yl)-4-methylmorpholinium chloride) (10 mM). Reaction between crosslinker and complex took place at room temperature for one hour. One microliter of 1 M ammonium bicarbonate was added and incubated for 15 min. at room temperature for quenching the cross-linking reaction. As there was no cysteine in the amino acid sequences of the complex, no reduction and alkylation steps were performed. Freshly prepared trypsin solution to an enzyme/protein ratio of ∼1:50 was added at 37 °c and allowed to incubate overnight. 10% (vol/vol) TFA solution to a final TFA concentration of 1% (vol/vol) was added to quench the enzymatic digestion. Before LC/MS, Micro spin columns (Harvard Apparatus) were used to desalt the sample using 5% acetonitrile, 0.1% TFA as washing solution, and 75% acetonitrile, 0.1% TFA as elution buffer.

Peptide mixtures were analyzed by LC- MS/MS on an UltiMate 3000 RSLC nano-HPLC system (Thermo Fisher Scientific) coupled to an Orbitrap Exploris 480 mass spectrometer (Thermo Fisher Scientific). Peptides were separated on reversed-phase C18 precolumn: (300 μm × 5 mm PepMap C18, Thermo Fisher Scientific) followed by a separation column Aurora (25 cm × 75 μm ID, 1.7 μm C18; IonOptiks). After online desalting, peptides were eluted and separated using a linear water-acetonitrile gradient from 5% to 30% B (with solvent B 99.9% (v/v) acetonitrile, 0.1% (v/v) formic acid) over 80 min.

Data were acquired in a data-dependent tandom mass spectrometry (MS/MS) mode with stepped higher-energy collision induced dissociation and normalized collision energies of 22%, 27%, and 30%. Each high-resolution full scan (*m/z* 350 to 1300, R = 60,000 at *m/z* 200) in the orbitrap was followed by 10 high-resolution product ion scans (R = 30,000), starting with the most intense signal in the full-scan mass spectrum (isolation window 2 *Th*). Precursor ions with charge states +1, +2, and nonassigned were excluded from fragmentation. Dynamic exclusion was enabled (duration 60 s). The acquired raw data were processed using Proteome Discoverer (version 3.0.0.400; https://www.thermofisher.com/fr/fr/home/industrial/mass-spectrometry/liquid-chromatography-mass-spectrometry-lc-ms/lc-ms-software/multi-omics-data-analysis/proteome-discoverer-software.html) with the integrated third-party XlinkX/PD nodes (53). Normal peptide search was performed using the Sequest node against a database containing common contaminants and the SEP3, AG, and PHYL_OY_ sequences (see PRIDE repository for the full FASTA database) *in silico* digested with Trypsin/P with a minimal peptide length of six and two miss cleaved sites allowed. Cysteine carbamidomethylation was set as fixed modification. Methionine oxidation and protein N-term acetylation were set as dynamic modifications. Filtering at 1% false discovery rate (FDR) at the peptide level was applied through the Percolator node. For cross-linked peptides, a database search was performed using XlinkX/PD nodes, with the same protease and dynamic modifications allowed but an increased number of missed cleavages of 3. Identified fragmentation spectra were accepted at an estimated FDR of maximum 1%. The resulting set was used to construct the list of unique crosslinks which was also FDR controlled at 1%. A final filter was applied where a minimal score of 39 and a minimal delta score of 4 was set. The crosslinks were replicate filtered (2 out of 3 independent cross-linking experiments), mapped and visualized onto the structure using XMAS (32) within ChimeraX (54). After this filtering step essentially very few to no false positives are expected to be present.

## Data availability

The mass spectrometry proteomics data have been deposited to the ProteomeXchange Consortium *via* the PRIDE (55) partner repository with the data set identifier PXD074375 and PXD074375. The SAXS data sets have been deposited in the SASBDB (56) under codes ID 7382, ID 7381, and ID 7374. AF3 models for Figures 1 and 4 are available from the ModelArchive under entries 10.5452/ma-00aks, 10.5452/ma-ai62r, and 10.5452/ma-yhix7.

## Supporting information

This article contains supporting information.

## Conflict of interest

The authors declare that they have no conflicts of interest with the contents of this article.
