# Supplementary material for: The phytoplasma effector, phyllogen, structurally mimics host plant MADS transcription factors[image]
Source: J Biol Chem. 2026 Aug 13;302(10):113449. doi: 10.1016/j.jbc.2026.113449 (PMC13579947; doi:10.1016/j.jbc.2026.113449)
Supplement: Supplementary file 1 [file mmc2.pdf]

Supplemental Figures 1-9 for Galien, et al., *The phytoplasma effector, Phyllogen, structurally mimics host plant MADS transcription factors.*

Supplemental Figure 1

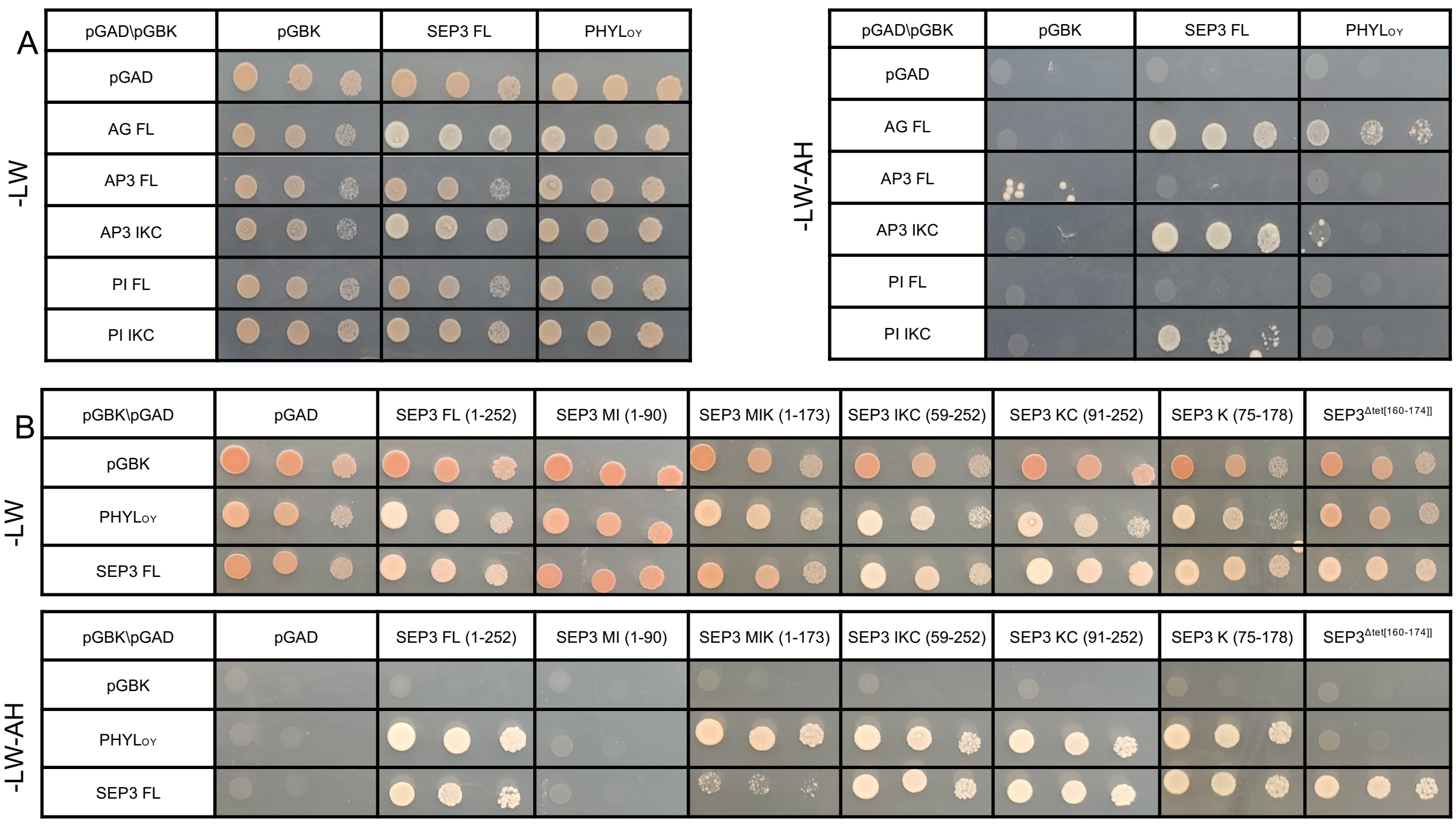

Supplemental Figure 1. Yeast 2-hybrid experiments for MTFs and PHYL$_{OY}$. A. At left, positive controls on media lacking leucine and tryptophan to confirm the presence of both vectors. At right, selective media demonstrating interactions for AG/SEP3, AG/PHYL$_{OY}$, AP3^{IKC}/SEP3 and PI^{IKC}/SEP3. No interactions were detected for PHYL$_{OY}$ with AP3^{IKC} or PI^{IKC}. AP3 and PI full length poorly interact with MTFs in yeast 2-hybrid, either due to poor expression or folding in the yeast system. B. Top, positive controls as per A. for Figure 1B for different SEP3 constructs. Bottom, PHYL$_{OY}$ requires the K domain of SEP3 for interaction (Figure 1B).

Supplemental Figure 2

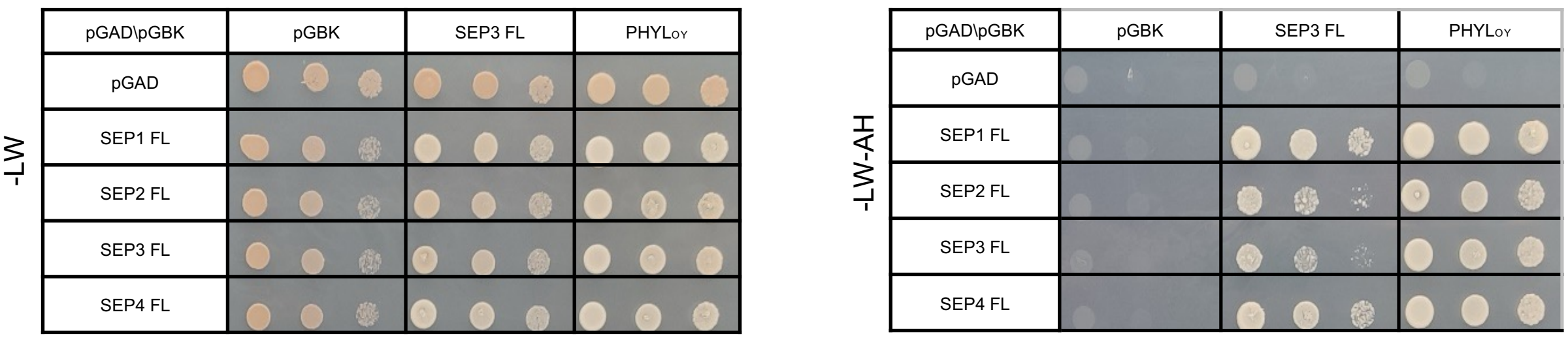

Supplemental Figure 2. Yeast 2-hybrid experiments for SEPs and PHYL$_{OY}$. Left, positive controls on media lacking leucine and tryptophan to confirm the presence of both vectors. Right, selective media demonstrating interactions for SEP1/SEP3, SEP2/SEP3, SEP3/SEP3, SEP4/SEP3, SEP1/PHYL$_{OY}$, SEP3/PHYL$_{OY}$ and SEP3/PHYL$_{OY}$ and SEP4/ PHYL$_{OY}$. pGAD and pGBK negative controls are the same as per Figure S1A (same plate). The SEP1, SEP2, SEP3 and SEP4 interactions (right panel) are shown in Figure 1A.

Supplemental Figure 3

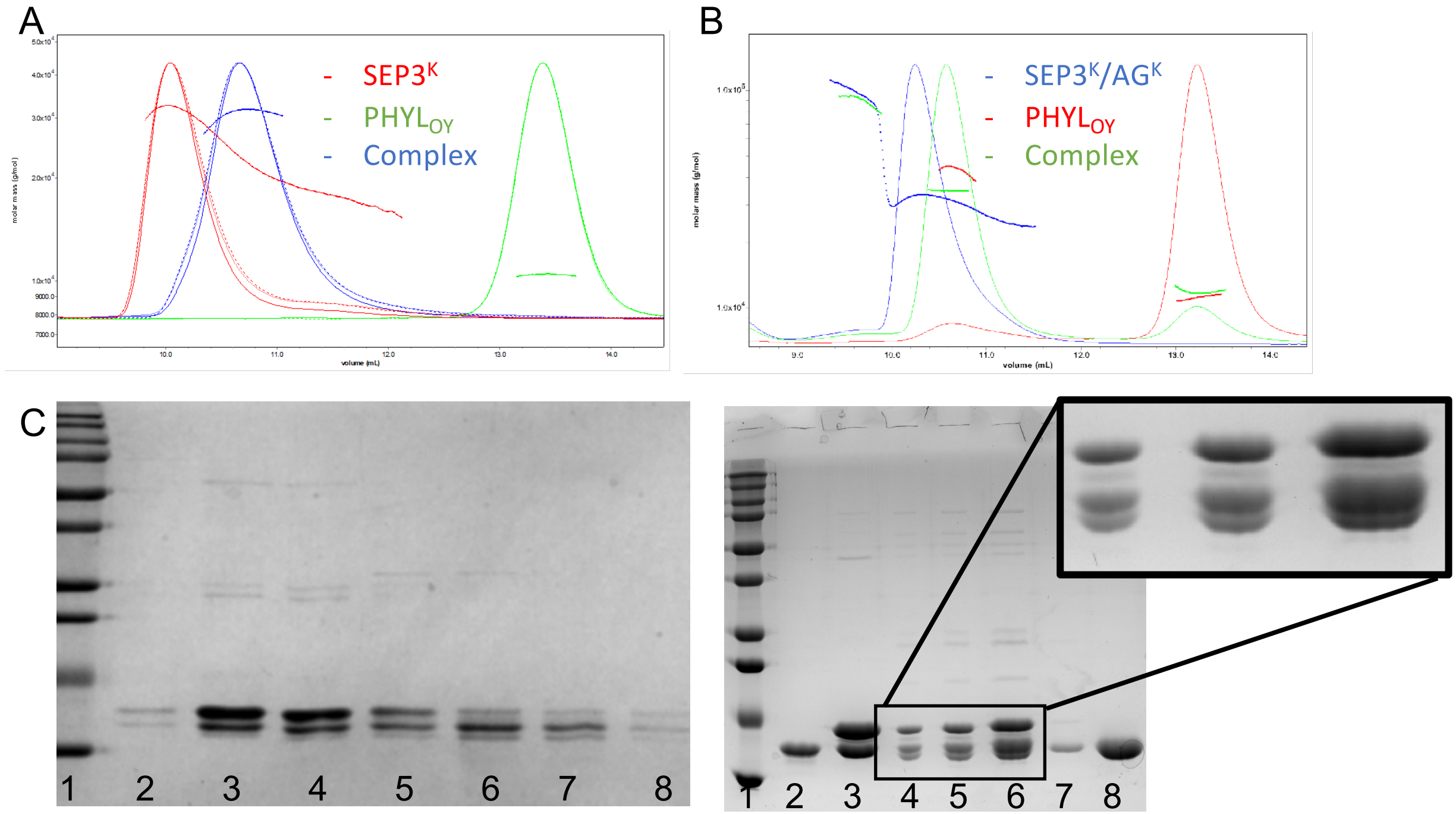

D

Experiment	Species	Elution volume (mL)	Theoretical MW (kDa)	Measured MW (kDa)	Stoichiometry
SEP3^K/PHYL$_{OY}$	SEP3^K	10.1	12 (monomer)	31.6	Dimer/tetramer equilibrium
	PHYL$_{OY}$	13.4	10.7 (monomer)	10.4	Monomer
	Complex	10.7	34.7 (SEP3^K/SEP3^K/PHYL$_{OY}$)	31	2:1 SEP3^K : PHYL$_{OY}$
SEP3^K/AG^K/ PHYL$_{OY}$	SEP3^K/AG^K	10.3	23.6 (dimer)	32.8	Dimer/tetramer equilibrium
	PHYL$_{OY}$	13.2	10.7	11.2	Monomer
	Complex	10.6	34.3 (SEP3^K/AG^K/PHYL$_{OY}$)	34.7	1:1:1 (SEP3^K:AG^K: PHYL$_{OY}$)

Supplemental Figure 3. Size exclusion and SEC multiangle laser light scattering data for SEP3^K, PHYL$_{OY}$, AG^K and complexes. A. SEP3^K, PHYL$_{OY}$ and the SEP3^K/ PHYL$_{OY}$ were examined. A new peak corresponding to the complex is evident when the two proteins were mixed. B. SEP3^K/ AG^K, PHYL$_{OY}$ and the SEP3^K/ AG^K/ PHYL$_{OY}$ complex were examined. A new peak corresponding to the complex is evident when the three proteins were mixed. C. *Left*, SDS PAGE (15%) with lane 1, molecular weight markers (the proteins have molecular weights between 10 and 15 kDa), lane 2-5 SEP3^K /PHYL$_{OY}$ complex with stoichiometry of ~2 SEP3^K :1 PHYL based on band intensities, lane 6-8 fractions corresponding to mainly PHYL$_{OY}$ alone. *Right*, molecular weight markers, lane 2 PHYL$_{OY}$ alone, lane 3 SEP3^K /AG^K complex, lanes 4-6 fractions corresponding to SEP3^K /AG^K /PHYL$_{OY}$ complex with a stoichiometry of ~1:1:1 based on band intensities, lanes 7-8 fractions from the peak with PHYL$_{OY}$. D. Elution volumes, calculated and measured molecular weights from A and B.

Supplemental Figure 4

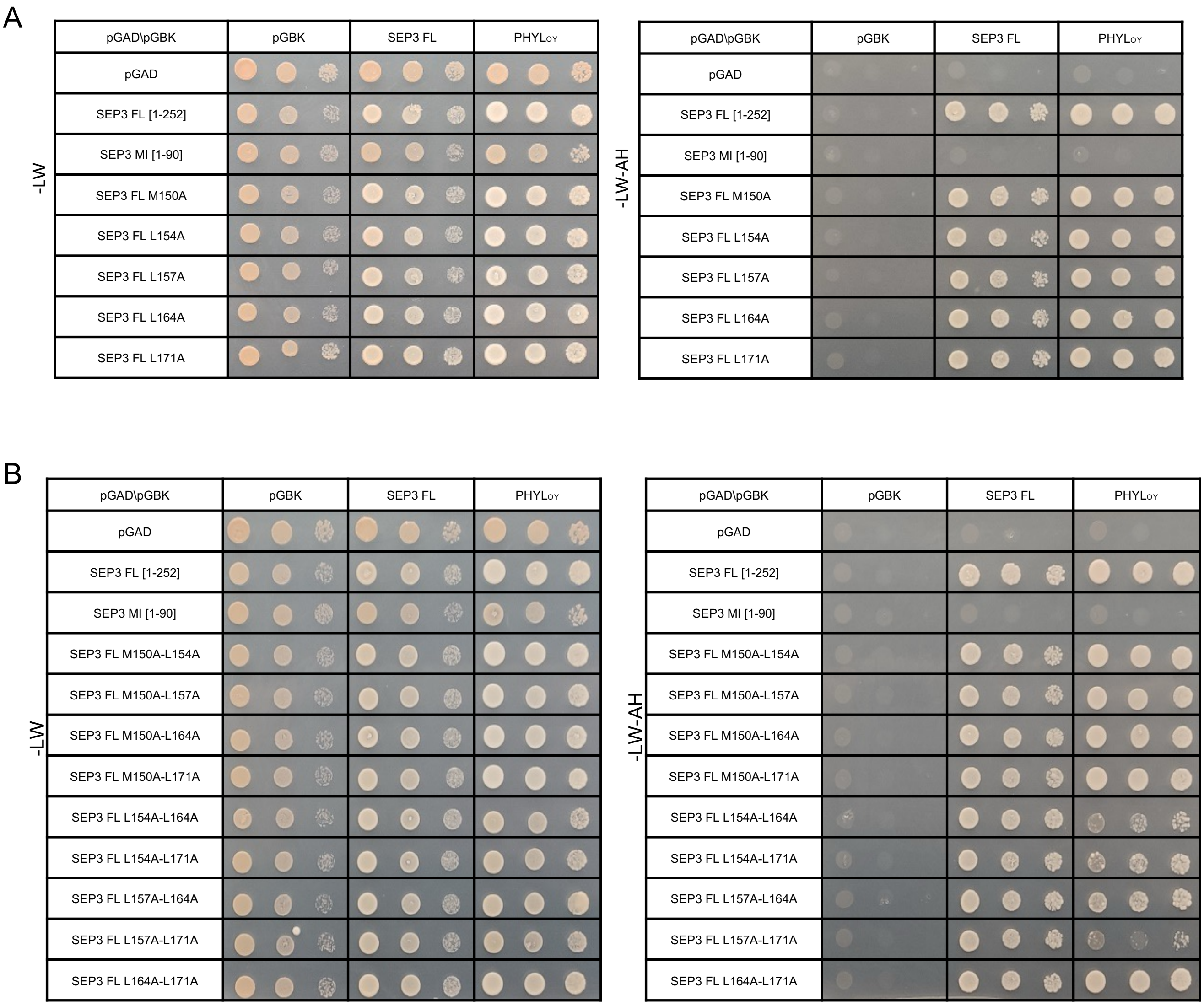

Supplemental Figure 4. Yeast 2-hybrid experiments for different SEP3 mutants and $PHYL_{OY}$. A. Left, control for the transformation with 2 vectors. Right, selective media demonstrating interactions for SEP3 single point mutations in the K domain and $PHYL_{OY}$. B. Left, control as per A. Right, double SEP3 mutants showing some attenuation of interaction with $PHYL_{OY}$. Control rows for SEP3 FL and $PHYL_{OY}$ and the SEP3 L164A, SEP3 L154A/L164A and SEP3 L157A/L171A mutant data are shown in Figure 3C.

Supplemental Figure 5

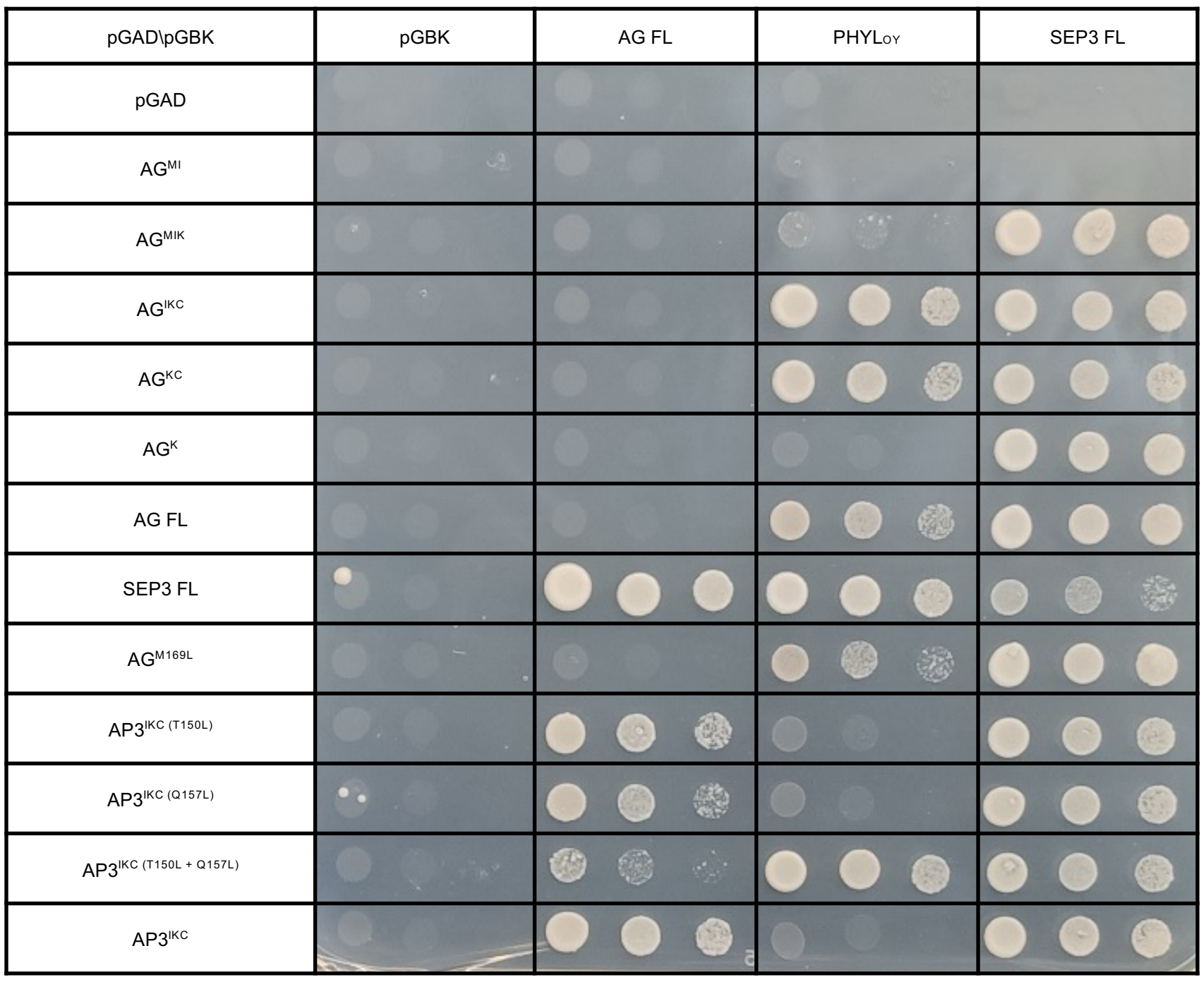

Supplemental Figure 5. Yeast 2-hybrid experiments for different MTF mutants and $PHYL_{OY}$. AG^{MI} (residues 1-102); AG^{K} (90-189); AG^{KC} (107-253); AG^{IKC} (74-253); AG^{MIK} (1-189); $AP3^{IKC}$ (59-233). The interactions (last four rows) between $PHYL_{OY}$ and $AP3^{IKC}$, AP3 T150L, Q157L and T150L/Q157L double mutant are shown in Figure 3D.

Supplemental Figure 6

A

Data-collection parameters		
Instrument:	Diamond light source B21	ESRF BM29
Wavelength (Å)	0.9524	0.99
q-range ($Å^{-1}$)	0.0026-0.34	0.007-0.5
Sample-to-detector distance (m)	3.7	2.6
Concentration range (mg/mL)	1-6	1-6
Temperature (K)	277-300	277-300
Detector	Eiger 4M (Dectris)	Pilatus 1M/Pilatus P3-2M *
Flux (photons/s)	$2*10^{12}$	$1.4*10^{12}/1*10^{13}$ *
Beam size (μm)	1102*240	700*700 /500*200 *
Absolute scaling method	NA	Comparison with scattering from 1 mm pure H_2O
Normalization	Transmitted intensity by beam-stop counter	Transmitted intensity by beam-stop counter
Monitoring for radiation damage	Frame-by-frame comparison	Frame-by-frame comparison
Exposure time	Continuous 2 s data-frame measurements of SEC elution	Continuous 1-3 s data-frame measurements of SEC elution

Structural parameters	$SEP3^{75-178}$	$SEP^{375-178}/AG^{90-189}$	$PHYL1_{OY}$	$SEP3^{75-178}/PHYL1_{OY}$	$SEP^{375-178}/AG^{90-189}$ $PHYL1_{OY}$
Data generatted from ScatterIV					
I0 (kDa) [from Guinier]	$1.63*10^{-1}$ +- $2.1*10^{-4}$	$1.68*10^{-1}$ +- $5.7*10^{-2}$	$3.70*10^{-2}$ +- $4.0*10^{-5}$	$7.93*10^{-2}$ +- $1.2*10^{-4}$	$1.21*10^{-1}$ +- $8.3*10^{-2}$
Rg (Å) [from Guinier]	40.2 +- 0.32	36.14 +- 0.75	21.8 +- 0.11	38.1 +- 0.37	33.39 +- 1.28
qminRg - qmaxRg used for Guinier ($Å^{-1}$)	0.295-1.30	0.01-0.036	0.304-1.30	0.274-1.30	0.014-0.039
qmin - qmax ($Å^{-1}$)	$2.4*10^{-4}$-$3.7*10^{-4}$	$9.4*10^{-3}$-$3.6*10^{-2}$	$1.4*10^{-2}$-$6.0*10^{-2}$	$7.2*10^{-3}$-$3.4*10^{-2}$	$1.3*10^{-2}$-$3.9*10^{-2}$
Volume ($Å^3$)	$5.3*10^5$	$2.6*10^5$	$5.1*10^4$	$2.0*10^5$	$1.9*10^5$
D_{max} (Å) (Moore Model)	154	112	66	136	101
Calculated MW from Vc (KDa)	55 (340)	33	18 (33)	51 (130)	20
Calculated theoretical MW monomer (kDa):	24.0 (dimer)	22.7	14.5	38.5	33.3
SEC-MALS	See Figure S2 C for all SEC-MALS data				

Software employed	
Primary data reduction:	BM29 autoprocessing pipeline, FREESAS (Kieffer 2021)
Data processing	Scatter IV (Tully 2021)
Forward models and ensemble fitting	FoXS (Schneidman-Duhovny, D 2010), Multi FoXS (Schneidman-Duhovny, D 2016), WAXSiS (Knight and Hub, 2015)

* Detector and flux for BM29 before and after ESRF upgrade to 4th generation source

B

C

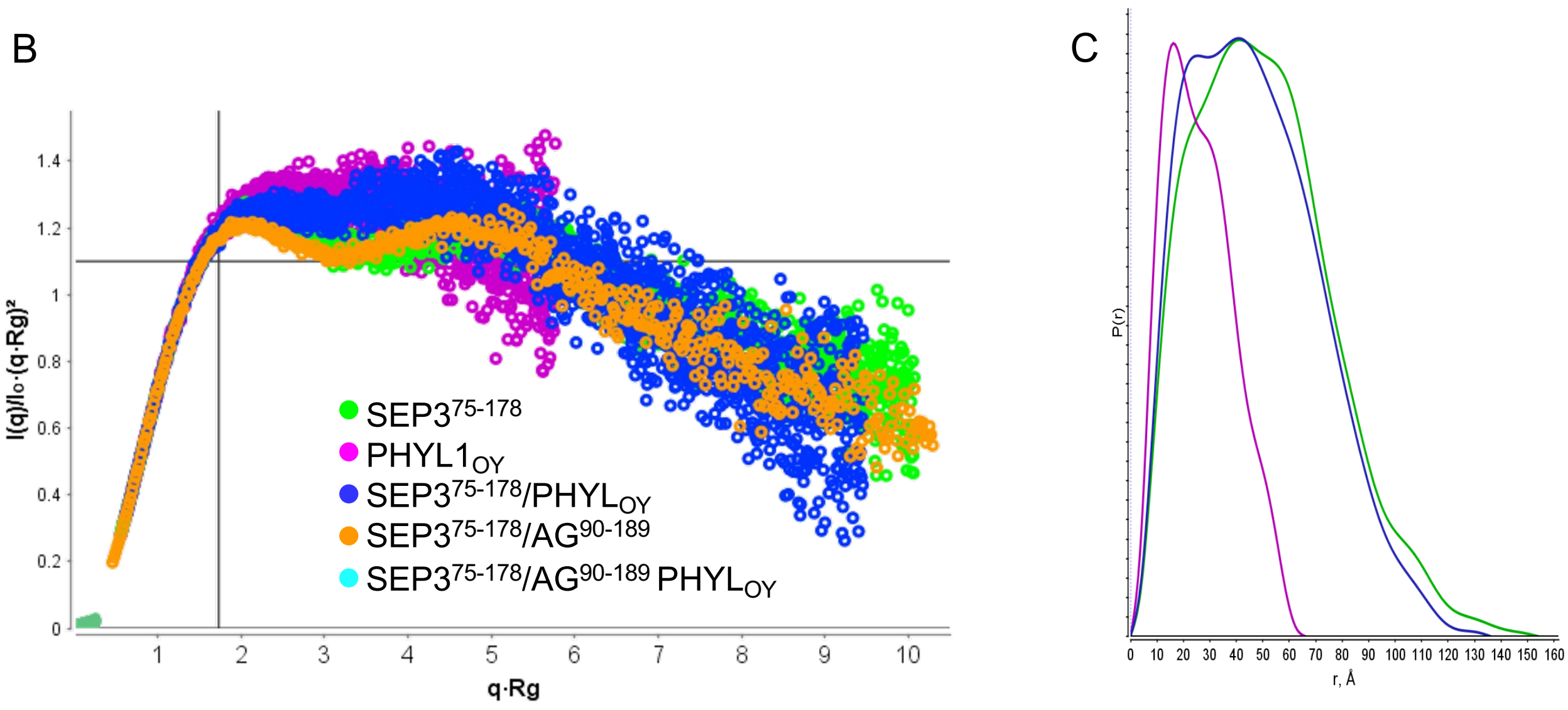

Supplemental Figure 6. SAXS data for MTF and $PHYL_{OY}$. A. SAXS data collection and analysis statistics. B. Kratky plot showing some flexibility of the proteins and complexes. C. P(r) pair distance distribution function indicative of elongated multidomain structures.

Supplemental Figure 7

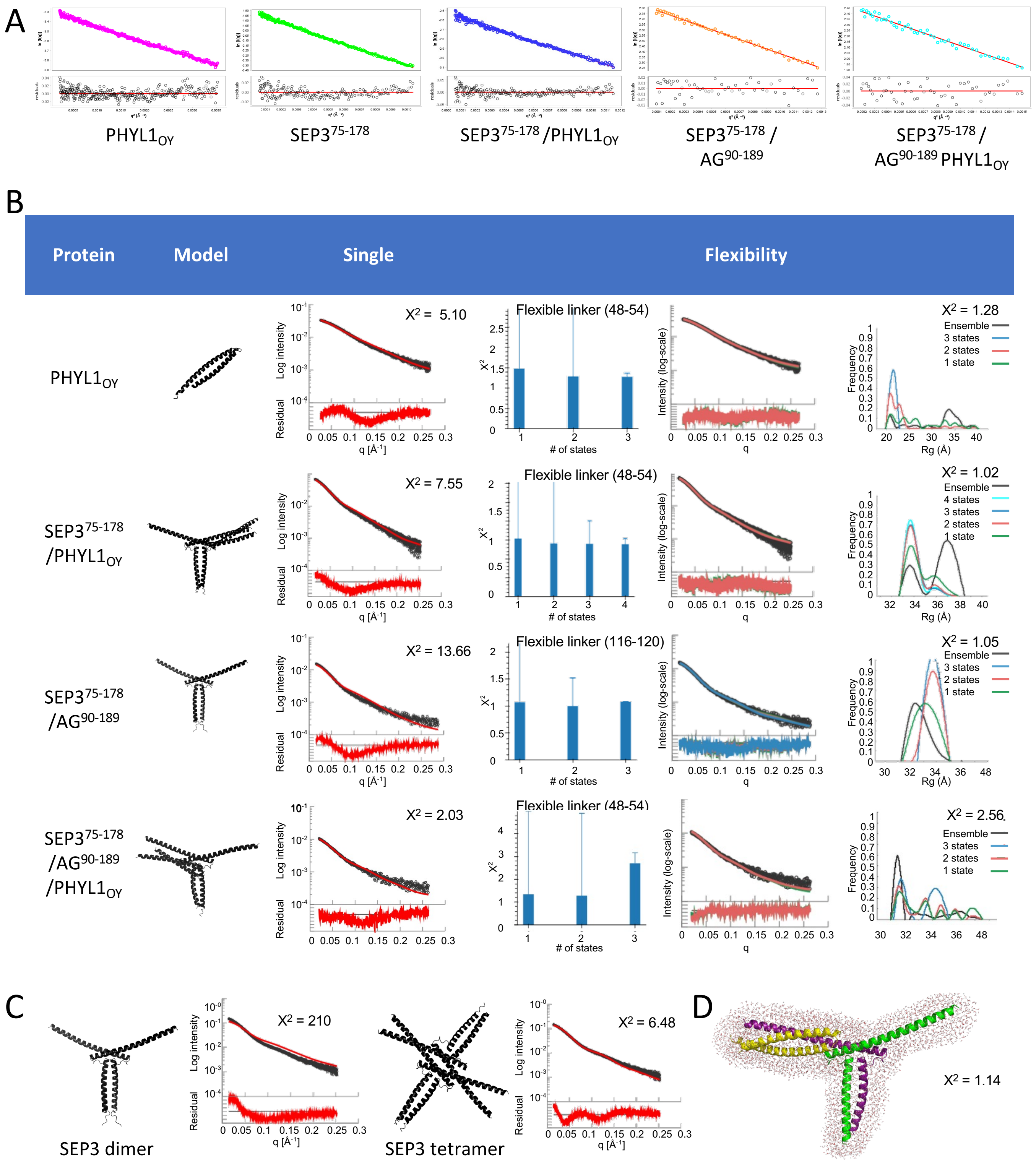

Supplemental Figure 7. SAXS data and model fitting. A. Guinier region showing linear fits for R_g calculations. B. Proteins and complexes fit with a single rigid model and with added flexibility using FoXS and multiFoXS. Chi^2 values are shown, with improvement for most fits to the experimental data with additional states due to flexibility in PHYL$_{OY}$. C. SEP3 dimer and tetramer models fit to experimental SAXS curves. D. WAXSiS model fit of SEP3$^{75\text{-}178}$ /AG$^{90\text{-}189}$ PHYL1$_{OY}$ to account for waters. Numbering corresponds to full length protein amino acid positions.

Supplemental Figure 8

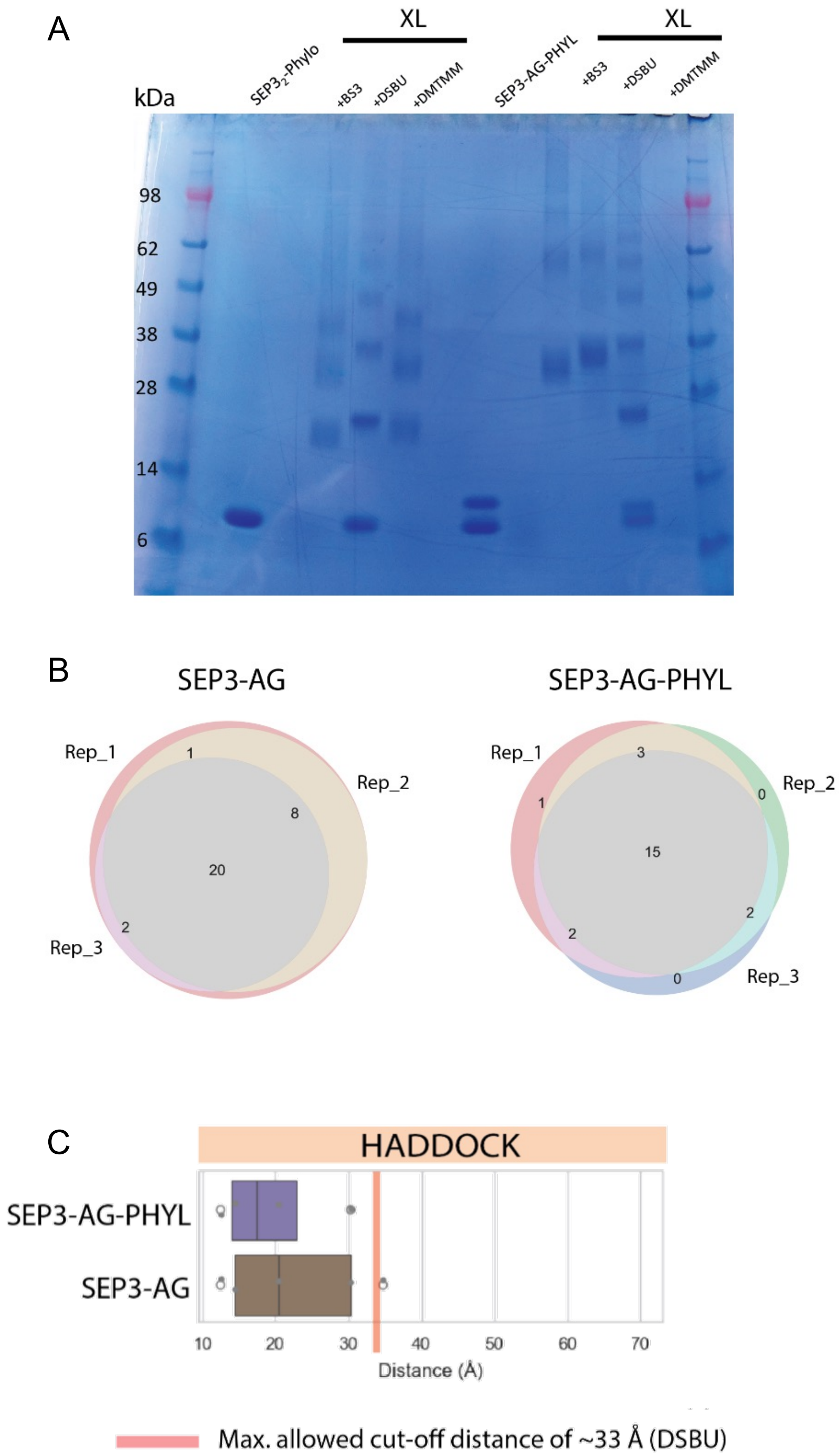

Supplemental Figure 8. Mass spectrometry experiments for $SEP3^K/PHYL_{OY}$ and $SEP3^K/AG^K/\ PHYL_{OY}$. A. Crosslinking SDS gel with DSBU. B. Three replicates gave high reproducibility for crosslinking. C. Crosslinks identified below a cut-off distance of ~33Å for SEP3/AG and $SEP3/AG/PHYL_{OY}$.

Supplemental Figure 9

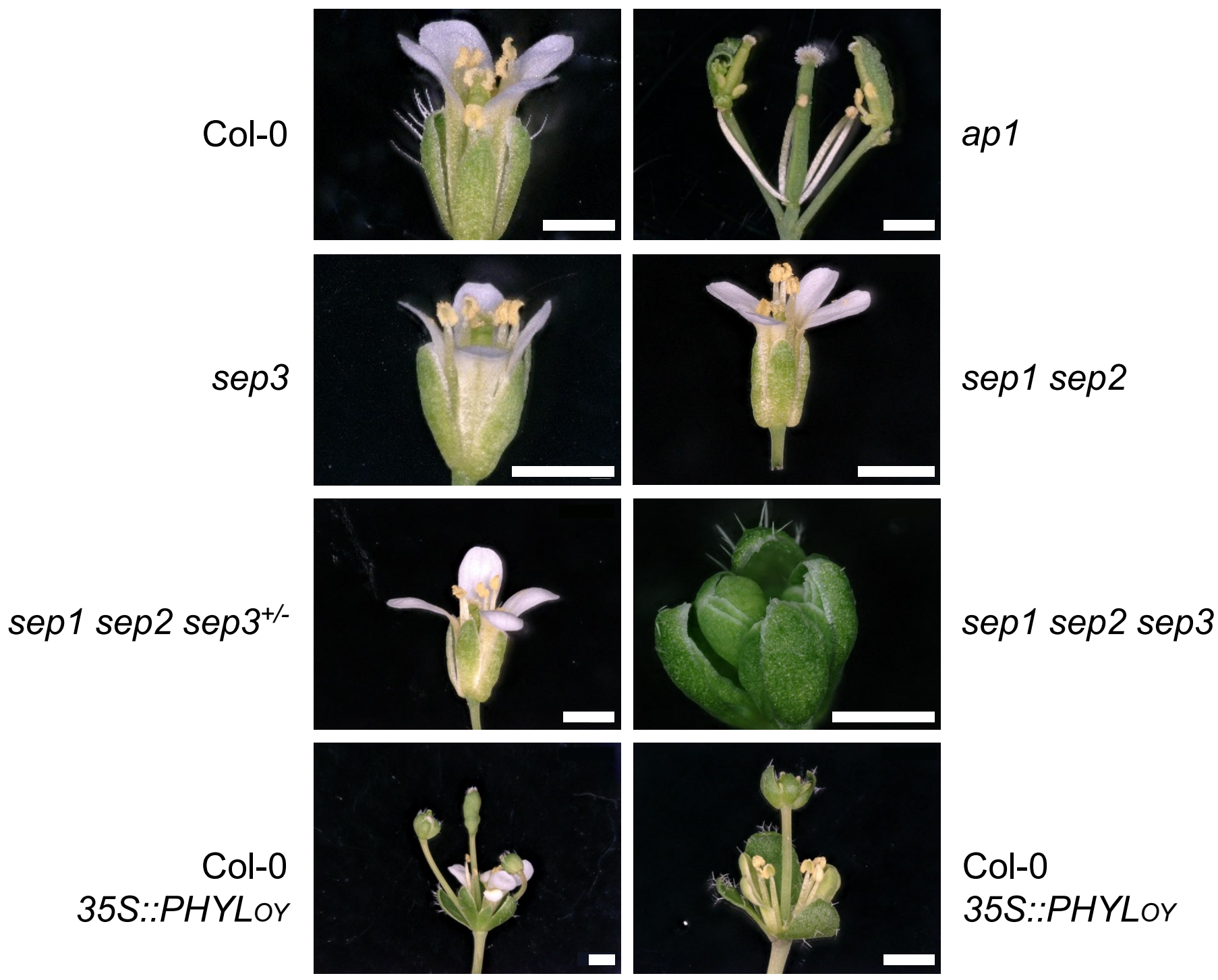

Supplemental Figure 9. Flower phenotypes of Arabidopsis WT and mutants used in this study. Col-0 (WT) with four sepals, four petals, six stamens and two fused carpels organised in whorls. The *ap1* mutant exhibits loss of organ identity in the first and second whorls with *ap1*-flower axillary buds in the second whorl. Third and fourth whorl development is normal. The *sep3, sep1 sep2, sep1 sep2 sep3*$^{+/-}$ show normal floral organ development. The triple mutant, *sep1 sep2 sep3,* shows sepaloid organs in all four whorls and indeterminacy of the fourth whorl giving rise to a sepaloid « flower-within-a-flower » phenotype. Col-0 plants expressing *35S:PHYL*$_{OY}$ show defects for 15/15 plants ranging from only first and fourth whorl defects (bottom, left, 4/15) to strong defects with all four whorls affected (bottom, right), consistent with published phenotypes. Col-0, top left panel, is shown as Col-0 in Figure 5A.
